# Glucosidase inhibitor, Nimbidiol ameliorates renal fibrosis and dysfunction in type-1 diabetes

**DOI:** 10.1038/s41598-022-25848-1

**Published:** 2022-12-15

**Authors:** Subir Kumar Juin, Sathnur Pushpakumar, Suresh C. Tyagi, Utpal Sen

**Affiliations:** Department of Physiology, University of Louisville School of Medicine, Louisville, KY 40202 USA

**Keywords:** Kidney diseases, Diabetes complications

## Abstract

Diabetic nephropathy is characterized by excessive accumulation of extracellular matrix (ECM) leading to renal fibrosis, progressive deterioration of renal function, and eventually to end stage renal disease. Matrix metalloproteinases (MMPs) are known to regulate synthesis and degradation of the ECM. Earlier, we demonstrated that imbalanced MMPs promote adverse ECM remodeling leading to renal fibrosis in type-1 diabetes. Moreover, elevated macrophage infiltration, pro-inflammatory cytokines and epithelial‒mesenchymal transition (EMT) are known to contribute to the renal fibrosis. Various bioactive compounds derived from the medicinal plant, *Azadirachta indica* (neem) are shown to regulate inflammation and ECM proteins in different diseases. Nimbidiol is a neem-derived diterpenoid that is considered as a potential anti-diabetic compound due to its glucosidase inhibitory properties. We investigated whether Nimbidiol mitigates adverse ECM accumulation and renal fibrosis to improve kidney function in type-1 diabetes and the underlying mechanism. Wild-type (C57BL/6J) and type-1 diabetic (C57BL/6‐*Ins2*^*Akita*^/J) mice were treated either with saline or with Nimbidiol (0.40 mg kg^−1^ d^−1^) for eight weeks. Diabetic kidney showed increased accumulation of M1 macrophages, elevated pro-inflammatory cytokines and EMT. In addition, upregulated MMP-9 and MMP-13, excessive collagen deposition in the glomerular and tubulointerstitial regions, and degradation of vascular elastin resulted to renal fibrosis in the Akita mice. These pathological changes in the diabetic mice were associated with functional impairments that include elevated resistive index and reduced blood flow in the renal cortex, and decreased glomerular filtration rate. Furthermore, TGF-β1, p-Smad2/3, p-P38, p-ERK1/2 and p-JNK were upregulated in diabetic kidney compared to WT mice. Treatment with Nimbidiol reversed the changes to alleviate inflammation, ECM accumulation and fibrosis and thus, improved renal function in Akita mice. Together, our results suggest that Nimbidiol attenuates inflammation and ECM accumulation and thereby, protects kidney from fibrosis and dysfunction possibly by inhibiting TGF-β/Smad and MAPK signaling pathways in type-1 diabetes.

## Introduction

The most common and detrimental pathophysiological complication of diabetes mellitus is diabetic nephropathy (DN) that has emerged as an alarming threat to the global population. DN is a chronic kidney disease (CKD) which is characterized by excessive accumulation and deposition of extracellular matrix (ECM) in the glomeruli and tubulointerstitium, progressing to renal fibrosis, deterioration of renal function, and eventually to end-stage renal disease (ESRD)^1–3^. Dysregulation of the major ECM proteins such as collagen, fibronectin, α-smooth muscle actin (α-SMA), and elastin is the hallmark of renal fibrosis and failure in DN^1,4,5^. Emerging evidence indicates that elevated infiltration and activation of macrophage, inflammation and myofibroblasts accumulation largely contribute to the development and progression of renal fibrosis^6,7^. Elevated macrophage infiltration is a common feature in renal fibrosis^8,9^. Activated macrophages synthesize different pro-inflammatory cytokines and chemokines, such as transforming growth factor-β1 (TGF-β1), tumour necrosis factor-α (TNF-α), interleukin-1β (IL-1β), and monocyte chemoattractant protein-1 (MCP-1). TNF-α is the crucial mediator of primary inflammatory response and plays an important role in DN^10^. TNF-α stimulates mesangial cells to produce MCP-1, which, in turn, promotes macrophage recruitment and progression of renal fibrosis^10–12^. Previous reports demonstrated that ECM deposition during renal fibrosis is predominantly contributed by the accumulation of myofibroblasts that are differentiated from macrophages, resident fibroblasts, epithelial cells, endothelial cells, pericytes etc.^13–15^. Epithelial‒mesenchymal transition (EMT) indicates the process of differentiation of the epithelial cells to the mesenchymal phenotype leading to the myofibroblast transdifferentiation and excessive interstitial collagen deposition^11,16–18^. Moreover, previous findings demonstrated that elevated α-SMA expression in tubular and glomerular epithelial cells is involved in the glomerulonephritis and glomerulosclerosis^16,19,20^. TGF-β1 is thought to play as a key mediator of EMT and acts as an important pro-fibrotic factor to promote excessive ECM accumulation leading to glomerulosclerosis and tubulointerstitial fibrosis in DN^13,21^.

Matrix metalloproteinases (MMPs) are important zinc-dependent endopeptidases that collectively control the synthesis and degradation of all ECM components including collagen and elastin^1,6^. Previous reports suggested that MMPs play critical role in the regulation of inflammatory response and progression of EMT^6^. MMP-9 has been shown to regulate inflammation in experimental glomerulonephritis in vivo^22^. MMP-9 was also reported to be a crucial regulator of EMT in murine renal tubular cells and renal interstitial fibrosis in obstructive nephropathy^23,24^. Previous studies demonstrated that elevated MMP-9 and MMP-13 cause adverse ECM remodeling in type-1 diabetic kidney^4,25,26^.

Nimbidiol is a diterpenoid derived from the root and stem-bark of the medicinal plant, *Azadirachta indica* (commonly known as ‘neem’) and is reported to possess potential anti-diabetic properties by inhibiting glucosidases^27^. Several previous studies exhibited antimicrobial, antioxidant, anti-inflammatory, anti-fibrotic, and anticancer potential of diverse bioactive compounds derived from neem^28,29^. A wide range of neem extracts and neem-derived compounds have also been reported to show antihyperglycemic activity^29^. Moreover, earlier studies have also suggested that bioactive compounds from *A. indica* regulate ECM proteins by modulating expression of MMPs in different diseases including wide-spectrum cancer studies^30–34^. Recently, therapeutic intervention of neem extract has been shown to abrogate inflammation-driven renal fibrosis by reducing ECM accumulation in UUO nephropathy^35^. However, this is not known whether neem-derived Nimbidiol modulates MMPs and ECM proteins in diabetic scenario. The present study investigated whether glucosidase inhibitor, Nimbidiol protects kidney from fibrosis and dysfunction in type-1 diabetes and the potential signaling mechanism.

## Results

### Effect of Nimbidiol on body weight, blood glucose level and α-glucosidase activity in the diabetic mice

**Figure 1:**
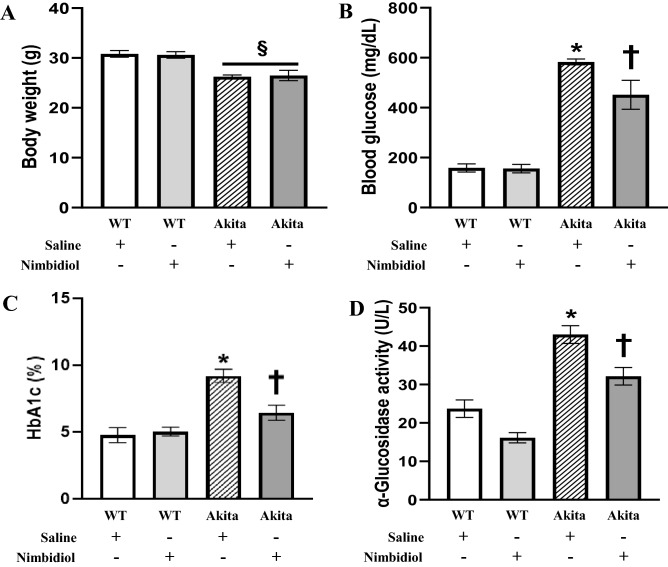
Effect of Nimbidiol on body weight, blood glucose level and α-glucosidase activity in the diabetic mice. The bar diagrams represent (**A**) body weight (**B**) blood glucose level (**C**) blood level of HbA1c and (**D**) α-glucosidase activity of the mice. Data are mean ± SD (n = 6/group). ^§^*p* < 0.05 versus WT + Saline and WT + Nimbidiol, *p < 0.05 versus WT + Saline, WT + Nimbidiol and Akita + Nimbidiol, †*p* < 0.05 versus WT + Saline, WT + Nimbidiol and Akita + Saline.

To evaluate the effect of Nimbidiol on body weights of the mice, all the mice were weighed at the end of the experiment. Akita mice showed significantly lower body weight compared to the age-matched WT mice (Fig. 1A). There was no significant change in body weight between WT mice treated with saline and Nimbidiol (Fig. 1A). Similarly, body weight of Nimbidiol-treated diabetic mice remained statistically unchanged compared to the diabetic mice that received saline (Fig. 1A). Previous reports suggested Nimbidiol as a promising antidiabetic drug due to its broad- spectrum glucosidase inhibition potentials^27,36^. Therefore, in the present study, we investigated whether Nimbidiol modulates levels of blood glucose, glycated haemoglobin (HbA1c) and α-glucosidase activity in type-1 diabetic mice. Our results showed significantly higher blood glucose and HbA1c levels in the Akita mice compared to that of WT control (Fig. 1B,C). Interestingly, blood glucose and HbA1c levels of the Akita mice treated with Nimbidiol were significantly reduced compared to the saline-treated diabetic mice (Fig. 1B,C). However, the blood glucose levels of Nimbidiol-treated Akita mice were higher than that of WT control (Fig. 1B). Our results also revealed a significant upregulation of the plasma α-glucosidase activity in Akita mice compared to the WT control (Fig. 1D). Notably, Nimbidiol treatment to Akita mice significantly reduced α-glucosidase activity compared to the saline-treated Akita mice (Fig. 1D).

### Nimbidiol treatment improved renal function in the diabetic mice

**Figure 2:**
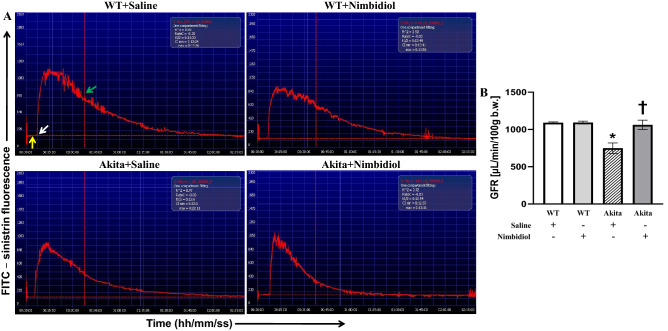
Nimbidiol treatment improved renal function in the diabetic mice. (**A**) Representative photographs of transcutaneous fluorescent emission of FITC-sinistrin for more than 2 h. ‘Yellow arrow’ indicates the background signal of the skin, ‘white arrow’ indicates time of injection of the FITC-sinistrin, and ‘green arrow’ indicates the time point selected to determine ‘R^2^’ and ‘t_1/2_’ used to calculate the ′GFR′ as mentioned in ‘Methods’. (**B**) The bar diagram represents GFR. Data are mean ± SD (n = 6/group). *p < 0.05 versus WT + Saline, WT + Nimbidiol and Akita + Nimbidiol, †*p* < 0.05 versus Akita + Saline.

Glomerular filtration rate (GFR) is considered as an important marker of renal function^37^. Previously, progressive decline in the GFR is reported in DN^38^. Therefore, to investigate the effect of Nimbidiol, renal function of the mice was evaluated by monitoring GFR. WT mice showed normal GFR (Fig. 2A,B). In diabetic mice, GFR was found to be significantly decreased compared to that of WT control (Fig. 2A,B). However, impaired renal function in diabetic mice was significantly improved with Nimbidiol treatment compared to the saline-treated Akita mice (Fig. 2A,B). There was no significant difference in GFR between saline-treated and Nimbidiol-treated WT mice (Fig. 2A,B).

### Renal cortical blood flow in the diabetic mice was normalized in response to Nimbidiol treatment

**Figure 3:**
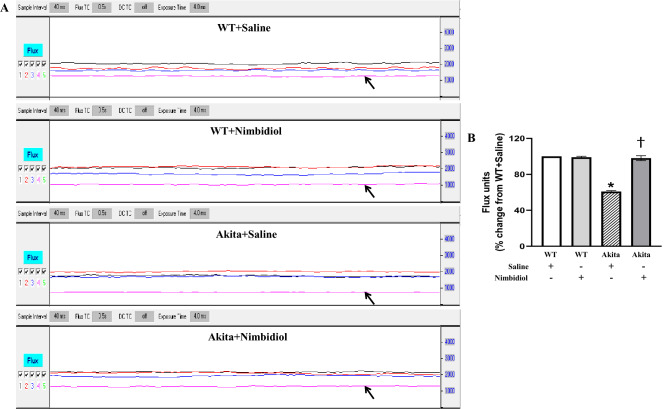
Renal cortical blood flow in the diabetic mice was normalized in response to Nimbidiol treatment. (**A**) Representative line tracing from laser Doppler flowmetry shows ‘flux units’ (no. of RBCs × velocity) in the aorta (black trace), renal artery (red trace), renal vein (blue trace), and renal cortex (pink trace, arrow). (**B**) The bar diagram represents the flux units as the percent change in the renal cortex versus WT + Saline. Data are mean ± SD (n = 6/group). *p < 0.05 versus WT + Saline, WT + Nimbidiol and Akita + Nimbidiol, ^†^*p* < 0.05 versus Akita + Saline.

In order to investigate whether Nimbidiol influences renal cortical blood flow, we performed laser Doppler flowmetry that serves as an efficient non-invasive method for evaluating intra-renal blood flow^39^. The results revealed that diabetic mice showed significantly (39%) reduced renal cortical blood flow compared to the basal level of the WT control (Fig. 3A,B). Compared to the saline-treated diabetic mice, treatment with Nimbidiol in diabetic mice significantly increased the renal cortical blood flow that was comparable to that of WT control (Fig. 3A,B). Renal cortical blood flow remained statistically unaltered between saline-treated and Nimbidiol-treated WT mice (Fig. 3A,B).

### Nimbidiol ameliorated resistive index (RI) of the renal cortical artery in the diabetic mice

**Figure 4:**
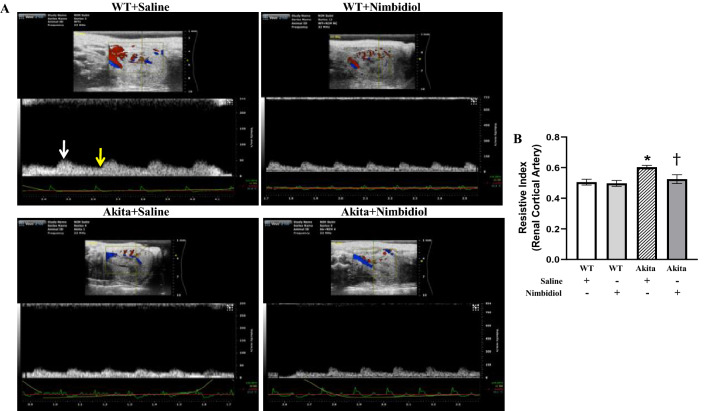
Nimbidiol ameliorated resistive index (RI) of the renal cortical artery in the diabetic mice. (**A**) Representative images from ultrasound of renal cortical artery. Resistive index was calculated using the formula: (PSV-EDV)/PSV. PSV, peak systolic velocity (white arrow); EDV, end diastolic velocity (yellow arrow). (**B**) The bar diagram represents the resistive index. Data are mean ± SD (n = 6/group). *p < 0.05 versus WT + Saline, WT + Nimbidiol and Akita + Nimbidiol, ^†^*p* < 0.05 versus Akita + Saline.

Resistive index (RI) indicates vascular elasticity and is considered as an important marker of kidney function^40^.To evaluate the effect of Nimbidiol on RI of the renal cortical artery, renal ultrasound was performed. Compared to the WT control, RI of the renal cortical artery in Akita mice was significantly increased, which was ameliorated by Nimbidiol treatment (Fig. 4A,B). However, Nimbidiol-treated WT mice showed no significant change in RI of the renal cortical artery compared to the WT mice receiving saline (Fig. 4A,B).

### Nimbidiol treatment normalized the expression of collagen IV, fibronectin and elastin, and mitigated histopathogical changes in the diabetic kidney

**Figure 5:**
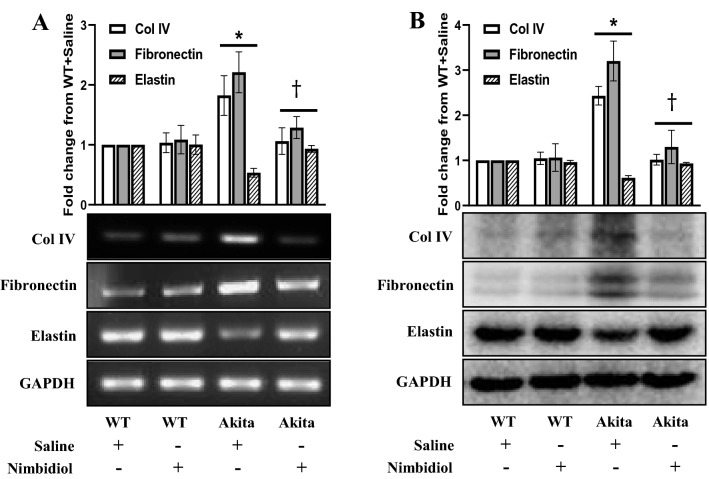
Nimbidiol treatment normalized the expression of collagen IV, fibronectin and elastin in the diabetic kidney. Representative images from (**A**) Semi-quantitative RT-PCR analyses showing gene expression and (**B**) western blot analyses showing protein expression of Col IV, fibronectin and elastin in the kidney. The bar diagrams represent the fold change from WT + Saline. Data are mean ± SD (n = 6/group). *p < 0.05 versus WT + Saline, WT + Nimbidiol and Akita + Nimbidiol, ^†^*p* < 0.05 versus Akita + Saline.

Adverse ECM accumulation in glomerulus and tubulointerstitium promotes renal fibrosis and subsequent deterioration of renal function^2^. ECM regulation by neem-derived compounds has already been shown in cancer studies^33,34^. Therefore, in our current study, we examined the effect of Nimbidiol on the expression of important ECM proteins such as collagen IV (Col IV), fibronectin, and elastin in the diabetic kidney. No significant difference in Col IV, fibronectin and elastin expression was observed between saline- and Nimbidiol-treated WT kidney both at mRNA and protein levels (Fig. 5A,B). Compared to the WT mice, diabetic kidney showed a robust upregulation of Col IV and fibronectin expression at mRNA (0.82 and 1.21 fold, respectively) and protein (1.43 and 2.20 fold, respectively) levels (Fig. 5A,B). Elastin expression in the kidney of diabetic mice was significantly downregulated at mRNA (0.47 fold) and protein (0.39 fold) levels compared to the WT mice (Fig. 5A,B). Nimbidiol treatment significantly downregulated the expression of Col IV and fibronectin, and upregulated elastin expression in diabetic kidney compared to the saline-treated Akita mice (Fig. 5A,B).

**Figure 6:**
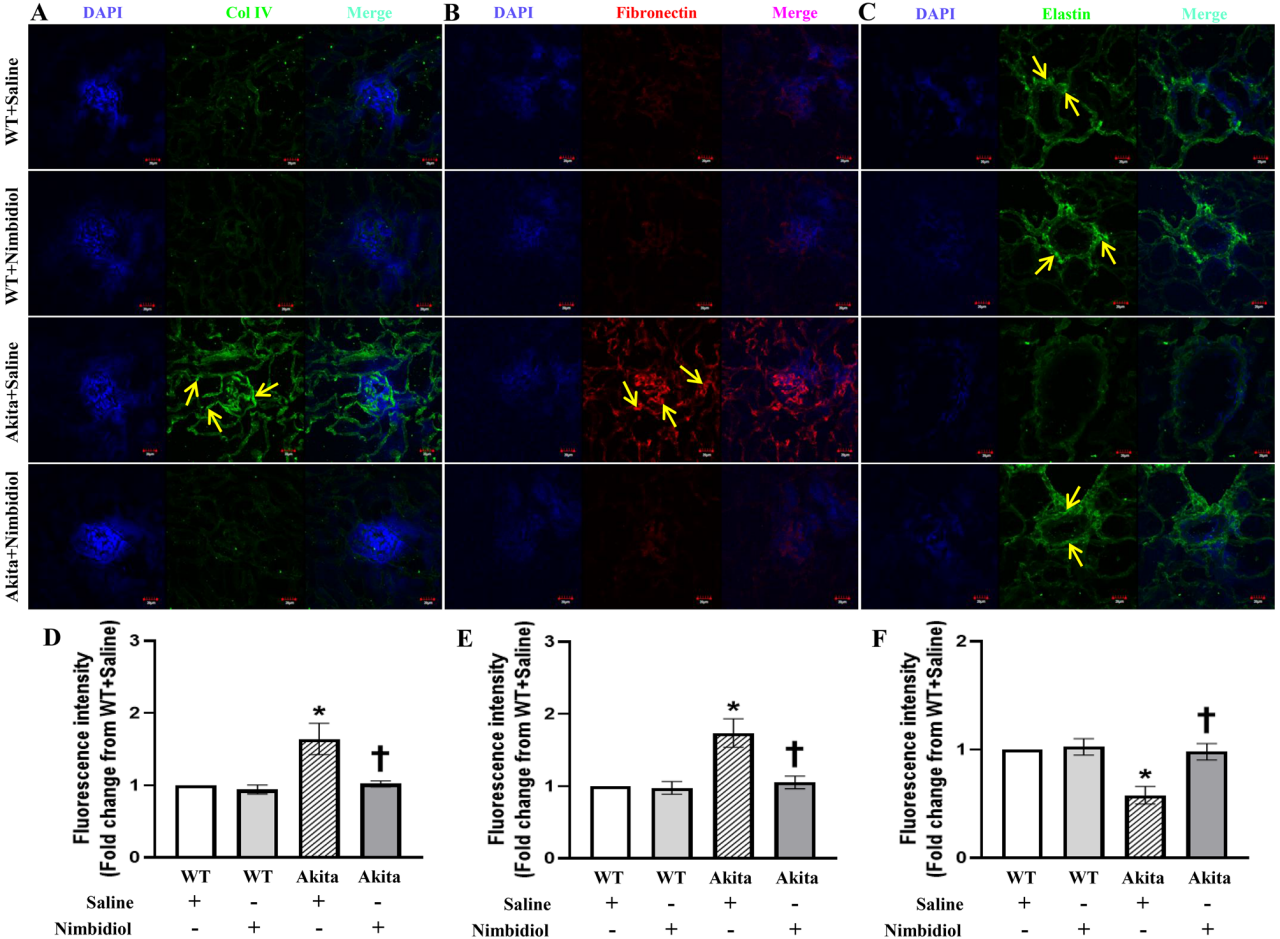
Nimbidiol inhibited upregulation of collagen IV and fibronectin, and downregulation of elastin expression in the diabetic kidney. Representative immunofluorescence images of the renal cortex showing expression of (**A**) Col IV, (**B**) fibronectin and (**C**) elastin. The nuclear counterstaining was performed using DAPI (blue). The bar diagrams represent the fold change in the fluorescence intensity from WT + Saline for **(D)** Col IV (**E**) fibronectin and (**F**) elastin. Data are mean ± SD (n = 6/group). *p < 0.05 versus WT + Saline, WT + Nimbidiol and Akita + Nimbidiol, ^†^*p* < 0.05 versus Akita + Saline. Scale bar: 20 µm; magnification × 60.

Consistent with the mRNA and western blot analyses, immunohistochemical localization revealed a sharp upregulation of Col IV and fibronectin in the glomerulus and tubulointerstitium, and a robust downregulation of vascular elastin expression in the interlobular artery of the diabetic mice compared to WT control (Fig. 6A‒F). Notably, Nimbidiol treatment to the diabetic mice significantly decreased Col IV and fibronectin expression and increased elastin expression compared to that of saline-treated Akita mice (Fig. 6A‒F). Compared to the saline-treated WT mice, expression of Col IV, fibronectin and elastin statistically unaltered in the kidney of the Nimbidiol-treated WT mice (Fig. 6A‒F).

**Figure 7:**
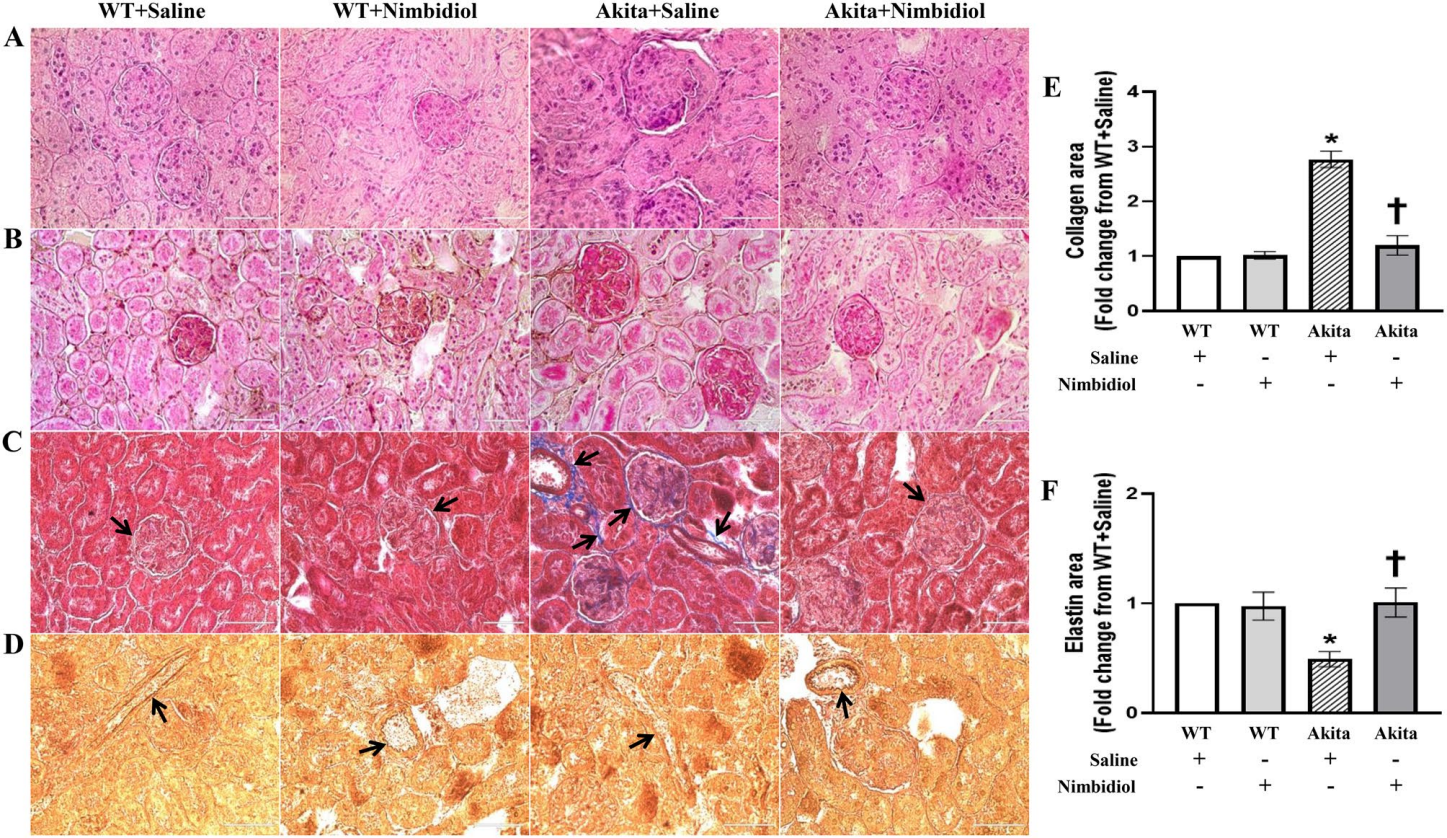
Nimbidiol mitigated histopathological changes in the diabetic kidney. Representative photomicrographs of kidney sections stained with (**A**) Hematoxylin and Eosin (H&E) and (**B**) Periodic Acid-Schiff (PAS) stains showing prominent glomerulopathy as well as tubulointerstitial injury in the diabetic kidney. (**C**) Masson’s trichrome staining showing glomerular and tubulointerstitial collagen deposition (blue, black arrows) and (**D**) Verhoeff’s Van Gieson staining showing degradation of vascular elastin (dark brown, black arrows) in the renal cortex of the diabetic kidney. The bar diagrams represent the fold change in the total (**E**) collagen and (**F**) elastin area from WT + Saline. Data are mean ± SD (n = 6/group). *p < 0.05 versus WT + Saline, WT + Nimbidiol and Akita + Nimbidiol, ^†^*p* < 0.05 versus Akita + Saline. Scale bar: 50 µm; magnification × 60.

In order to monitor the potential histological changes, kidney sections were subjected to Hematoxylin and Eosin (H&E), and Periodic Acid-Schiff (PAS) staining. Kidney section of the Akita mice showed distinct glomerulopathy as well as tubulointerstitial injury as evidenced by prominent expansion and accumulation of mesangial matrix forming early nodules, thickening of the glomerular and tubular basement membrane, tubular dilation, interstitial widening etc. (Fig. 7A,B). Of note, Nimbidiol treatment ameliorated the histopathological changes in Akita mice (Fig. 7A,B). Further, Masson’s trichrome staining revealed a sharp increase in collagen deposition in the periglomerular, glomerular, and also tubulointerstitial regions of the diabetic kidney compared to the WT mice (Fig. 7C,E). It was interesting to observe that Nimbidiol treatment to Akita mice substantially decreased collagen deposition in the kidney to the basal level that was comparable to the of WT control (Fig. 7C,E). There was no significant difference in the glomerular and tubulointerstitial collagen deposition between saline- and Nimbidiol-treated WT mice (Fig. 7C,E). To evaluate the elastin content of the renal cortical blood vessels, kidney sections were stained with Verhoeff′s Van Gieson stain. Vascular elastin content between saline- and Nimbidiol-treated WT mice remained statistically unaltered (Fig. 7D,F). Diabetic kidney showed a drastic degradation in elastin content compared to the WT control (Fig. 7D,F). Nimbidiol treatment to Akita mice substantially improved the elastin content that was comparable to the WT control (Fig. 7D,F).

### Nimbidiol attenuated epithelial‒mesenchymal transition (EMT) in the diabetic kidney

**Figure 8:**
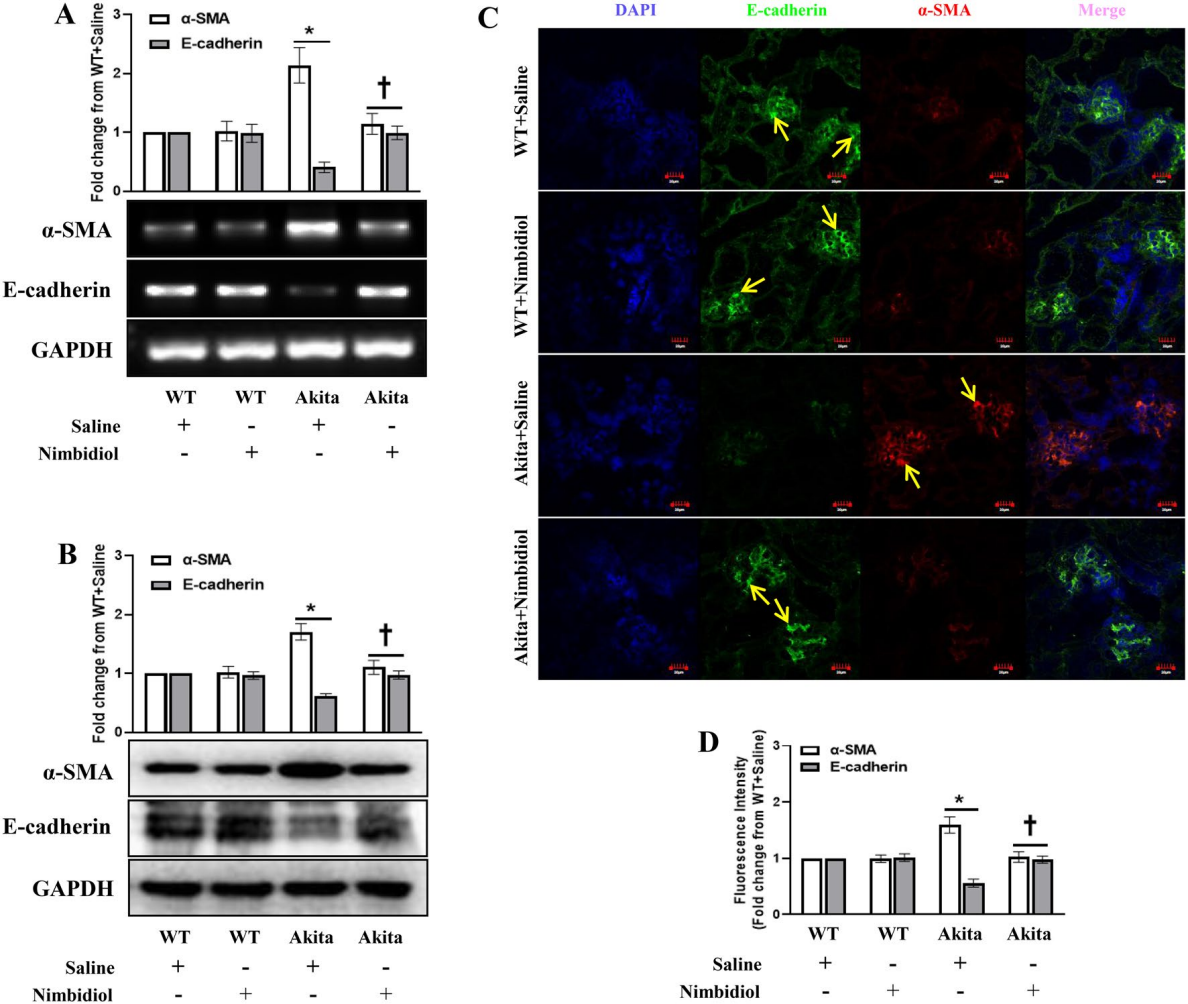
Nimbidiol attenuated epithelial‒mesenchymal transition (EMT) in the diabetic kidney. Representative images from (**A**) Semi-quantitative RT-PCR analyses showing gene expression and (**B**) western blot analyses showing protein expression of E-cadherin and α-SMA in the kidney. (**C**) Representative immunofluorescence images of the renal cortex demonstrate the expression of E-cadherin (green) and α-SMA (red). The nuclear counterstaining was performed using DAPI (blue). The bar diagrams represent the fold change in (**A**) gene expression, (**B**) protein expression and (**D**) fluorescence intensity from WT + Saline. Data are mean ± SD (n = 6/group). *p < 0.05 versus WT + Saline, WT + Nimbidiol and Akita + Nimbidiol, ^†^*p* < 0.05 versus Akita + Saline. Scale bar: 20 µm; magnification × 60.

Epithelial‒mesenchymal transition (EMT) is widely considered as a crucial mediator of glomerulosclerosis and tubulointerstitial fibrosis^11,19,41^. Therefore, we analyzed the expression of E-cadherin (epithelial marker) and α-SMA (mesenchymal marker) in the kidney and tested if Nimbidiol regulates their expression. Results revealed that compared to the WT control, diabetic kidney showed a significant downregulation of E-cadherin and upregulation of α-SMA expression at mRNA (0.59 and 1.14 fold, respectively) and protein (0.38and 0.71 fold, respectively) levels (Fig. 8A,B). Nimbidiol treatment normalized their expression in diabetic mice (Fig. 8A,B). The expression of E-cadherin and α-SMA remained statistically unaltered between WT mice treated with saline and Nimbidiol (Fig. 8A,B). Further, immunohistochemical staining exhibited a sharp downregulation of E-cadherin expression and a robust upregulation of α-SMA expression in the kidney of the diabetic mice compared to the WT control (Fig. 8C,D). Nimbidiol treatment restored basal level expression of E-cadherin and α-SMA in the diabetic kidney (Fig. 8C,D). No significant difference was observed in the expression of E-cadherin and α-SMA between saline- and Nimbidiol-treated WT mice (Fig. 8C,D).

### Nimbidiol reduced elevated expression of MMP-9 and MMP-13 in the diabetic kidney

**Figure 9:**
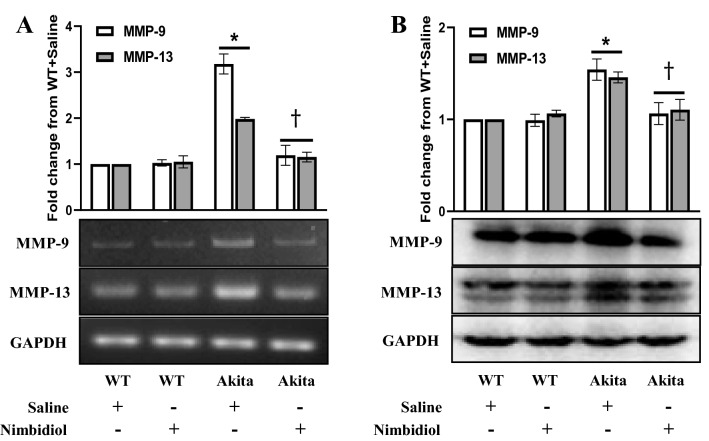
Nimbidiol reduced elevated expression of MMP-9 and MMP-13 in the diabetic kidney. Representative images from (**A**) Semi-quantitative RT-PCR analyses showing gene expression and (**B**) western blot analyses showing protein expression of MMP-9 and MMP-13 in the kidney. The bar diagrams represent the fold change from WT + Saline. Data are mean ± SD (n = 6/group). *p < 0.05 versus WT + Saline, WT + Nimbidiol and Akita + Nimbidiol, ^†^*p* < 0.05 versus Akita + Saline.

Deregulation of MMPs is an important driver of adverse ECM turnover leading to renal fibrosis^4,6,26^. Elevated expression of MMP-9 and MMP-13 was reported to be associated with renal fibrosis in DN^25,42,43^. Previously, neem-derived compounds have been shown to modulate MMP-9 expression in cancer^31,32^. Therefore, we investigated the effect of Nimbidiol on the expression of MMP-9 and MMP-13 in the diabetic kidney. The mRNA and protein expression of MMP-9 and MMP-13 in WT control remained at basal levels (Fig. 9A,B). There was no significant difference in the mRNA and protein expression of MMP-9 and MMP-13 between saline- and Nimbidiol-treated WT mice (Fig. 9A,B). Of note, compared to WT, diabetic kidney showed a significant increase in the expression of MMP-9 and MMP-13 at mRNA (2.18 and 0.98 fold, respectively) and protein (0.54 and 0.46 fold, respectively) levels (Fig. 9A,B). Nimbidiol treatment mitigated the elevated expression of MMP-9 and MMP-13 in the diabetic kidney (Fig. 9A,B).

### Accumulation of M1 macrophages in the diabetic kidney was attenuated by Nimbidiol

**Figure 10:**
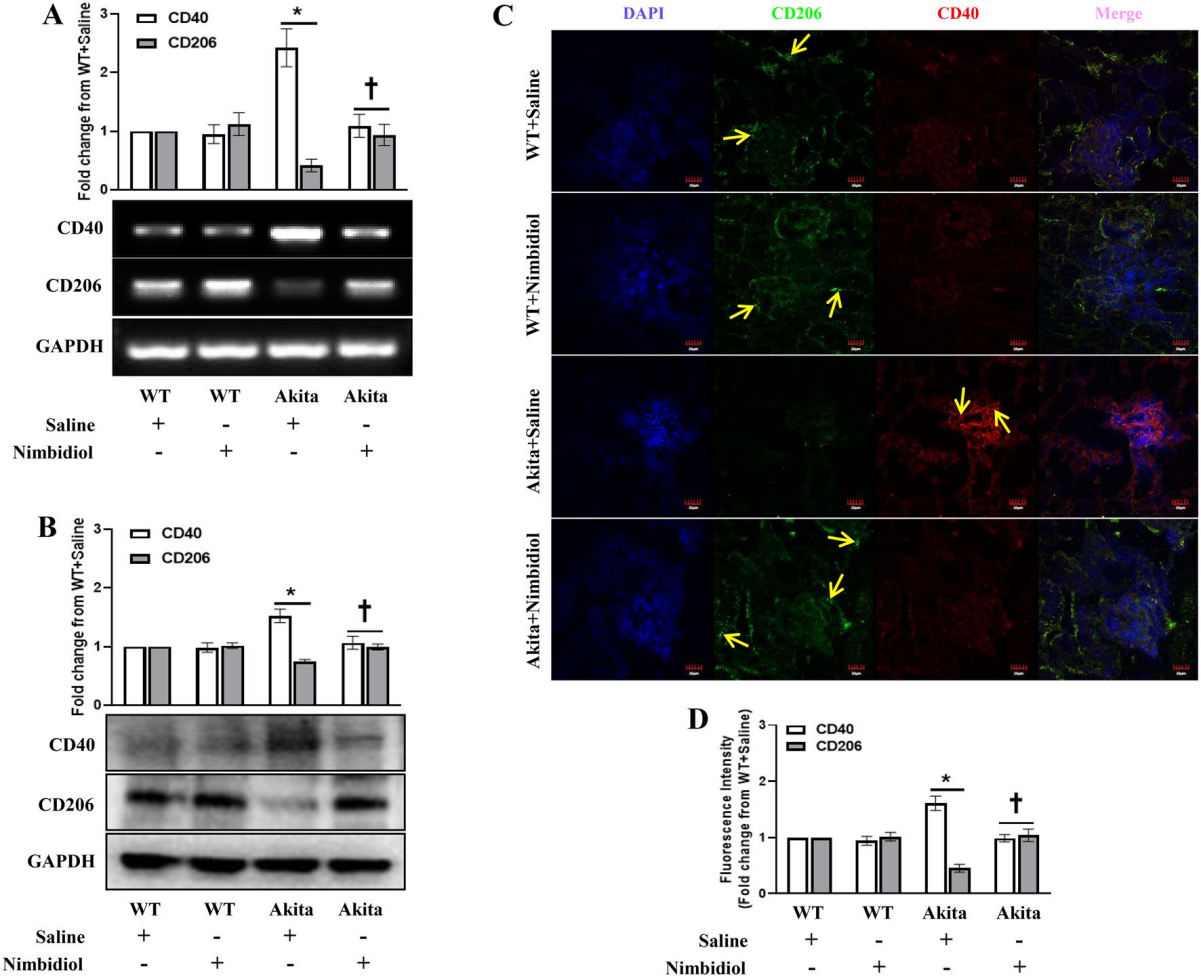
Accumulation of M1 macrophages in the diabetic kidney was attenuated by Nimbidiol. Representative images from (**A**) Semi-quantitative RT-PCR analyses showing gene expression and (**B**) western blot analyses showing protein expression of CD40 and CD206 in the kidney. (**C**) Representative immunofluorescence images of the renal cortex demonstrate the expression of CD206 (green) and CD40 (red). The nuclear counterstaining was performed using DAPI (blue). The bar diagrams represent the fold change in (**A**) gene expression, (**B**) protein expression, and (**D**) fluorescence intensity from WT + Saline. Data are mean ± SD (n = 6/group). *p < 0.05 versus WT + Saline, WT + Nimbidiol and Akita + Nimbidiol, ^†^*p* < 0.05 versus Akita + Saline. Scale bar: 20 µm; magnification × 60.

Macrophages are the crucial mediators of renal inflammation and fibrosis in different renal diseases including DN^11,14^. Increased infiltration of pro-inflammatory M1 macrophages in the glomerulus and interstitium is highly involved in the initiation and progression of renal fibrosis in DN^11^. On the other hand, reparative roles of anti-inflammatory M2 macrophages have been shown in different kidney diseases including UUO and diabetic nephopathy^11,44,45^. Therefore, we investigated whether Nimbidiol modulates macrophage accumulation in the diabetic mice. In diabetic kidney, CD40 was upregulated and CD206 was downregulated significantly both at mRNA (1.42 and 0.59 fold, respectively) and protein (0.39and 0.26 fold, respectively) levels compared to the WT mice (Fig. 10A,B). Nimbidiol treatment normalized their expression in the diabetic mice (Fig. 10A,B). There was no significant difference in the expression of CD40 and CD206 between saline- and Nimbidiol-treated WT mice both at mRNA and protein levels (Fig. 10A,B). Similarly, immunohistochemical study revealed a robust increase of the CD40 + (pro-inflammatory M1-type) macrophages and a distinct decrease of the CD206 + (anti-inflammatory M2-type) macrophages in the kidney of the diabetic mice (Fig. 10C,D). However, the CD40 + and CD206 + macrophages in the kidney of the diabetic mice treated with Nimbidiol remained at the basal levels as observed in WT mice (Fig. 10C,D). There were no significant changes of the CD40 + and CD206 + macrophages between saline- and Nimbidiol-treated WT mice (Fig. 10C,D).

### Nimbidiol treatment mitigated elevated pro-inflammatory cytokine and chemokine in the diabetic kidney

**Figure 11:**
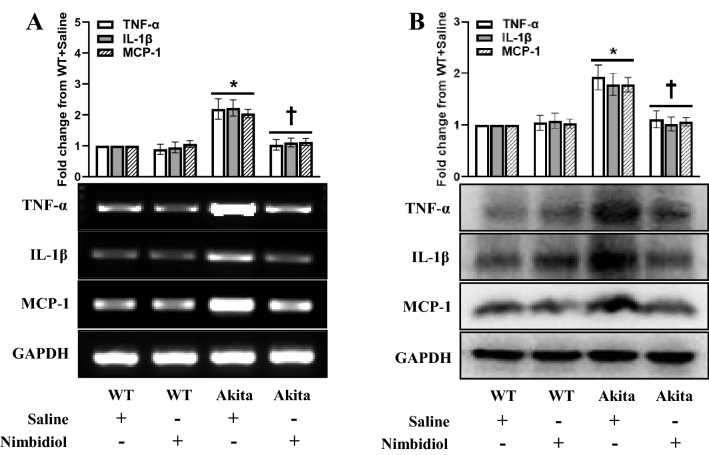
Nimbidiol treatment mitigated elevated pro-inflammatory cytokine and chemokine in the diabetic kidney. Representative images from (**A**) Semi-quantitative RT-PCR analyses showing gene expression and (**B**) western blot analyses showing protein expression of TNF-α, IL-1β and MCP-1 in the kidney. The bar diagrams represent the fold change from WT + Saline. Data are mean ± SD (n = 6/group). *p < 0.05 versus WT + Saline, WT + Nimbidiol and Akita + Nimbidiol, ^†^*p* < 0.05 versus Akita + Saline.

A plethora of evidence suggests that inflammation plays a pivotal role in the development and progression of DN, while pro-inflammatory cytokines and chemokines remain crucial mediators of inflammation^11,46^. Therefore, the present study investigated the expression of different pro-inflammatory cytokine and chemokine such as TNF-α, IL-1β and MCP-1 in the kidney, and tested whether Nimbidiol modulates their expression. The expression of TNF-α, IL-1β and MCP-1 was significantly increased at mRNA (1.19, 1.22 and 1.04 fold, respectively) and protein (0.93, 0.79 and 0.78 fold, respectively) levels in the diabetic mice compared to that of WT control (Fig. 11A,B). Notably, Nimbidiol treatment to diabetic mice reduced their expression to the basal levels that were comparable to the WT mice (Fig. 11A,B). WT mice treated with Nimbidiol showed no significant difference in the expression of TNF-α, IL-1β and MCP-1 compared to that of saline-treated WT mice (Fig. 11A,B).

### Nimbidiol inhibited TGF-β1, Smad2/3 and mitogen-activated protein kinases (MAPKs) in the diabetic kidney

**Figure 12:**
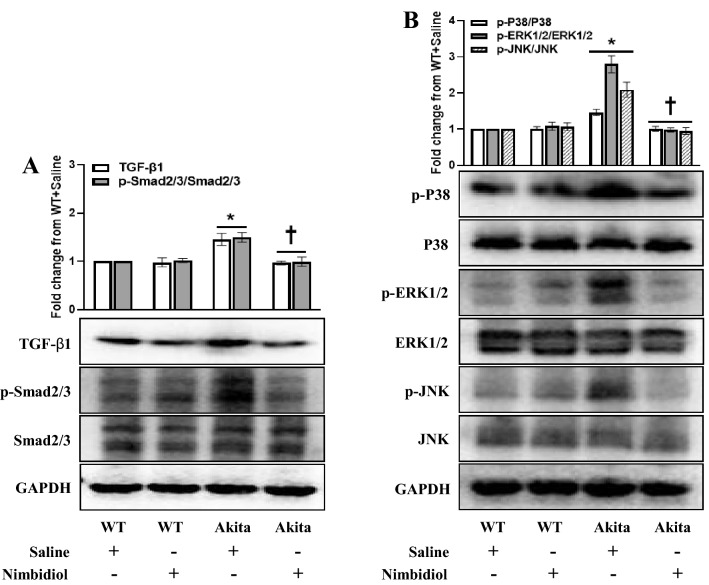
Nimbidiol inhibited TGF-β1, Smad2/3 and mitogen-activated protein kinases (MAPKs) in the diabetic kidney. Representative images from western blot analyses showing protein expression of (**A**) TGF-β1, Smad2/3 and p-Smad2/3, and (**B**) P38, p-P38, ERK1/2, p-ERK1/2, JNK and p-JNK in the kidney. The bar diagrams represent the fold change from WT + Saline. Data are mean ± SD (n = 6/group). *p < 0.05 versus WT + Saline, WT + Nimbidiol and Akita + Nimbidiol, ^†^*p* < 0.05 versus Akita + Saline.

TGF-β1/Smad and mitogen-activated protein kinase (MAPK) signaling pathways play critical roles in excessive ECM production and development of renal fibrosis^5,11,47^. Therefore, we evaluated the protein expression of TGF-β1, Smad2/3 and MAPKs (P38, ERK1/2 and JNK), and examined whether Nimbidiol modulates their expression in the kidney. Results showed that protein expression levels of TGF-β1 and phosphorylated- Smad2/3, P38, ERK1/2 and JNK were significantly (0.45, 0.50, 0.46, 1.80 and 1.09 fold respectively) upregulated in diabetic kidney compared to the WT mice (Fig. 12A,B). Nimbidiol treatment to the diabetic mice significantly reduced protein expression of TGF-β1 and phosphorylation of Smad2/3, P38, ERK1/2 and JNK to the basal levels, comparable to the WT control (Fig. 12A,B). Compared to the saline-treated WT mice, the expression of TGF-β1, p-Smad2/3, p-P38, p-ERK1/2 and p-JNK remained statistically unaltered in WT mice treated with Nimbidiol (Fig. 12A,B).

## Discussion

Diabetes mellitus (DM) is a complex metabolic disorder characterized by impaired glucose metabolism leading to hypergycemia i.e., elevated blood sugar level. Untreated DM often leads to diabetic nephropathy (DN) that refers to renal fibrosis, progressive deterioration of renal function, renal failure and ultimately to end-stage renal disease (ESRD). Treating DN has become a significant challenge as the prevalence of DN-related mortality and morbidity is increasing worldwide. Different extracts from *A. indica* (neem) have earlier been reported to show anti-diabetic properties by inhibiting glucosidases and reducing blood glucose levels^29,48–51^. Although different studies have elucidated the anti-diabetic potential of several neem-derived bioactive compounds, their role in DN remains unknown. The present study investigated the effect of Nimbidiol, a potent glucosidase inhibitor from the root/stem-bark of *A. indica*, on the pathophysiological complications in type-1 DN. Our study showed that in type-1 diabetic kidney, polarization of macrophage towards M1-type, elevated pro-inflammatory cytokine, epithelial-mesenchymal transition (EMT), imbalanced MMPs and adverse ECM accumulation result to renal fibrosis and deterioration of renal function as evidenced fromdecreased glomerular filtration rate (GFR). The changes were associated with increased resistance in the renal cortical artery and decreased renal cortical blood flow. Nimbidiol treatment decreased elevated M1 macrophage, pro-inflammatory cytokine and ECM accumulation to ameliorate renal fibrosis and improve arterial resistance and blood flow of the renal cortex and renal function via downregulation of TGF-β1, p-Smad2/3, p-P38, p-ERK1/2 and p-JNK in type-1 diabetic kidney.

In the present study, body weight of diabetic mice was found to be significantly lower than that of WT control, which was in accordance with the previous study^52^. Nimbidiol showed no effect on body weight. Previous studies reported that hyperglycemia acts as the central regulator in the pathogenesis of DN^1,53^. Our results revealed a robust increase in blood glucose level in Akita mice, which is a typical feature of type-1 diabetic Akita mice^54^. Nimbidiol treatment reduced blood glucose level in diabetic mice suggesting its hypoglycaemic property. Alpha-glucosidase inhibitors (AGIs) competitively inhibit glucosidases and thus decelerate complex carbohydrate catabolism and delay glucose synthesis leading to amelioration of overall diabetic health^36^. Nimbidiol is reported to reversibly inhibit the activities of sucrase-isomaltase, maltase-glucoamylase, lactase, trehalase and microbial α-glucosidases^27^. Inhibition kinetics of Nimbidiol on a wide-spectrum glucosidase was described earlier^27^. Our finding for the first time showed that Nimbidiol efficiently reduced α-glucosidase activity in Akita mice confirming that anti-hyperglycemic effect of Nimbidiol is due to its glucosidase inhibitory activity as presumed previously^27^. Renal resistive index (RI), renal blood flow and GFR are some of the pivotal physiological parameters that serve as crucial indicators of renal function and prognostic markers of DN^4,37,39,55^. Our study demonstrated that increased resistance and reduced blood flow in the renal cortex were associated with reduced GFR, indicating deteriorated renal function in type-1 diabetic mice, which was in accordance with the previous findings^4^. Our current study further showed that Nimbidiol treatment to Akita mice reduced renal cortical arterial resistance, increased renal cortical blood flow and thus, improved renal function, suggesting a renoprotective role of Nimbidiol in type-1 DN. In chronic kidney disease (CKD), increased RI and poor renal function were found to be associated with adverse histopathological changes and renal fibrosis^56^. In Akita mice it has also been demonstrated that elevated RI and decline in renal function were associated with adverse ECM accumulation^4^. Collagen and fibronectin are crucial ECM proteins excessive accumulation of which was reported to be associated with pathological complications of DN^57,58^. On the other hand, degradation of elastin, another important ECM protein is known be involved in renovascular remodeling during DN. In our present study, a sharp increase in collagen IV and fibronectin expression and a decrease in elastin expression were evidenced in diabetic kidney, similar to our previous studies^1,25^. Moreover, our study showed a robust collagen deposition in the glomerular and tubulointerstitial regions and degradation of vascular elastin content resulted to renal fibrosis in diabetic kidney, corroborating our earlier findings^1,4,25^. Further, we observed that Nimbidiol treatment normalized the expression of collagen IV, fibronectin and elastin in diabetic kidney. Moreover, Nimbidiol substantially reduced glomerular and tubulointerstitial collagen deposition and also attenuated vascular elastin degradation, indicating an important role of Nimbidiol in improving renal function in type-1 DN by ameliorating adverse ECM-induced fibrovascular pathology.

Epithelial‒mesenchymal transition (EMT) is characterized by the loss of E-cadherin and activation of α-SMA–positive myofibroblasts, which is known to trigger excessive ECM accumulation^59^. Notably, our present study showed a downregulation of epithelial marker, E-cadherin and upregulation of mesenchymal marker, α-SMA indicating EMT as an important contributor of excessive ECM accumulation in the type-1 diabetic kidney. It is worth mentioning that active participation of EMT to promote renal fibrosis has previously been shown in UUO nephopathy and also in type-1 DN^18,19,59–62^. Our study showed that Nimbidiol treatment normalized the expression of E-cadherin and α-SMA in diabetic kidney indicating that Nimbidiol mitigates ECM accumulation by inhibiting EMT and can be used as a potential anti-fibrotic agent in type-1 DN.

Matrix metalloproteinases (MMPs) are profoundly implicated in the ECM regulation in DN^63^. Involvement of elevated MMP-9 in excessive renal ECM accumulation has been evidenced in previous studies wherein elevated MMP-9 was shown incapable of degrading nonenzymatically glycated collagen IV leading to thickening of glomerular basement membrane^64,65^. Further,MMP-9 was reported to promote EMT and subsequent tubulointerstitial collagen accumulation^66–68^.The current study showed a sharp upregulation of MMP-9 and MMP-13 in diabetic kidney, which was found to be involved in excessive ECM accumulation and renal fibrosis, corroborating the previous findings^1,4,25,69,70^. It was noteworthy that the degree of increase in the mRNA expression of MMP-9 and MMP-13 was not reflected in the protein levels. The poor correlation between mRNA expression and protein levels has been explained earlier^71–73^. Cis- and trans-acting mechanisms, secondary structures of RNA, codon bias, ribosome density and occupancy, amino acid usage, post-transcriptional mechanisms and half-lives of protein, untranslated RNA species, secreted proteins, mRNA distribution and sequestration to the nucleus are some of the important factors that greatly influence quantitative correlations between mRNA expression and protein abundance levels^71–73^. As MMPs-9 and -13 are also secreted proteins thus it is highly plausible to observe such poor correlation between their mRNA and protein levels. Whatsoever, our study further showed that Nimbidiol treatment remarkably alleviated the elevated expression of MMP-9 and MMP-13 in diabetic kidney, indicating MMP regulation by Nimbidiol help mitigating adverse ECM remodeling in type-1 DN. Notably, previous studies have provided evidence of MMP-9 inhibitory role of multiple neem extracts in different diseases^30–32,74–76^. However, to the best of our knowledge, our current study for the first time showed an important role of Nimbidiol in regulation of MMPs, and thus, ECM accumulation in DN.

Macrophage infiltration and activation promotes continuous secretion a wide range of profibrogenic factors, inflammatory cytokines and chemokines that in turn, induce renal fibrosis in DN^11,14,24,47^.Moreover, several earlier studies reported that TNF-α, IL-1β and MCP-1 play crucial role in ECM accumulation and renal fibrosis^5,77–79^. Our present study showed that macrophage polarization towards M1-type, and increased pro-inflammatory cytokines and chemokine (TNF-α, IL-1β, MCP-1) were associated with renal fibrosis indicating evidence of a crucial role of macrophage mediated inflammation in the renal fibrosis and dysfunction in DN. Interestingly, Nimbidiol treatment attenuated macrophage polarization towards M1-type and cytokines upregulation resulting to the amelioration of diabetes induced renal inflammation and fibrosis in Akita mice. Of note, various neem extracts and neem-derived bioactive compounds have been reported to show potential role on macrophage polarization and anti-inflammatory activity in different diseases^74–76,80^. Our study provides strong evidence of anti-inflammatory role of Nimbidiol by macrophage repolarization in type-1 DN.

TGF-β1 is an important pro-fibrotic factor which plays a central role in renal inflammation and development of renal fibrosis^13,81^. TGF-β1 has been reported to promote renal fibrosis by various mechanisms such as direct synthesis of ECM proteins like collagen and fibronectin, mesangial cell proliferation, progression of EMT, depletion of podocytes and tubular epithelial cells etc. ^82–85^. It has been also reported that TGF-β1 inducesMMP-9, which in turn, promotes EMT and renal fibrosis ^66–68^. A plethora of evidence suggests that TGF-β1 primarily acts via intracellular signaling cascades such as Smads and MAPKs (P38, ERK1/2 and JNK) to promote EMT and renal fibrosis in CKD^84,86–88^. TGF-β1 phosphorylates Smad2/3, and p-Smad2/3 regulates transcription of the genes involved in fibrosis^89,90^. Previous studies demonstrated the potential of neem-derived bioactive compound in mitigating inflammation-induced fibrosis by reducing EMT and ECM deposition through inhibition of TGF-β/Smad signaling in different murine disease models including UUO nephropathy^35,91,92^. A wide-spectrum cancer studies have shown that neem extract plays an important role in the regulation of MAPKs^28^. Moreover, inhibition of MAPK phosphorylation by neem-derived compound has also been reported in earlier studies^93,94^. In our present study, normalization of TGF-β1, p-Smad2/3, p-P38, p-ERK1/2 and p-JNK in Nimbidiol-treated diabetic mice suggested that Nimbidiol may mitigate renal fibrosis in type-1 DN by attenuating TGF-β1/Smad and MAPK signaling pathways. Chronic hyperglycemia, oxidative stress, and advanced glycation end products (AGEs) are some of the crucial factors that stimulate TGF-β to promote renal fibrosis in the progression and development of DN^95,96^. A plethora of evidence showed that hyperglycemia or high glucose acts as the key regulator of TGF-β production and activation of MAPKs both in vitro and in vivo including DN^95–98^. As our study clearly exhibited that Nimbidiol alleviates hyperglycemia by inhibiting α-glucosidase activity thus, anti-hyperglycemic effect of Nimbidiol could play a crucial role to ameliorate renal fibrosis and dysfunction in type-1 diabetes. However, we agree that further studies are required to unravel the mechanistic insight into Nimbidiol action and whether Nimbidiol directly acts on the signaling molecules of TGF-β/Smad and/or MAPK pathway to regulate diabetic renal fibrosis and dysfunction in future research.

To summarize, hyperglycemia triggers accumulation of M1 macrophages along with elevated pro-inflammatory cytokine, chemokine and pro-fibrotic factors (TNF-α, IL-1β, MCP-1 and TGF-β1), that may be contributing to the increased α-SMA and decreased E-cadherin expression indicating EMT, and elevated levels of MMP-9 and MMP-13 expression in the diabetic kidney. Together, this leads to the upregulation of Col IV and fibronectin along with excessive collagen deposition in the glomerular and tubulointerstitial regions, and degradation of vascular elastin resulting to the renal fibrosis. These pathological changes were further associated with elevated renal cortical resistive index, reduced renal cortical blood flow and decreased GFR. Nimbidiol treatment reduced macrophage-mediated inflammation and elevated expression of TGF-β1, p-Smad2/3, p-P38, p-ERK1/2 and p-JNK leading to the amelioration of adverse ECM accumulation and improvement of renal function in type-1 diabetic mice. In conclusion, our study demonstrates that glucosidase inhibitor, Nimbidiol ameliorates renal fibrosis and dysfunction in type-1 diabetes possibly by inhibiting TGF-β/Smad and MAPK signaling pathways and therefore, Nimbidiol may be developed as a promising antidiabetic drug in future.

## Methods

### Reagents and chemicals

Fibronectin (cat. no. ab2413; 1 µg ml^−1^); andTGF-β1 (cat. no. ab64715; 2 µg ml^−1^) were from Abcam (Cambridge, CA, UK). α-SMA (cat. no. 19245S; 1:1000), E-cadherin (cat. no. 14472S; 1:1000), Smad2/3 (cat. no.8685S; 1:1000), p-Smad2/3 (cat. no. 8828S; 1:1000), P38 (cat. no.8690S; 1:1000), p-P38 (cat. no.9211S; 1:1000), ERK1/2 (cat. no. 4695P; 1:1000), p-ERK1/2 (cat. no.4370P; 1:2000), JNK (cat. no.9252P; 1:1000) and p-JNK (cat. no.4668P; 1:1000), antibodies were purchased from Cell Signaling Technology (Danvers, MA, USA). TNF-α (cat. no.60291-1-IG; 1:2500) antibody was from Proteintech (Rosemont, IL, USA). Collagen IV (cat. no. NBP1-26549; 1:1000) antibody was from Novus Biologicals LLC (Centennial, CO, USA). MMP-9 (cat. no. MA5-15886; 1:1000), MMP-13 (cat. no. 701287; 2 µg ml^−1^), MCP-1 (cat. no. MA5-17040; 1:1000) and fluorescently conjugated secondary antibodies, anti-rabbit (Alexa Fluor 594, cat. no. A-11012; 2 µg ml^−1^), anti-mouse (Alexa Fluor 488, cat. no. A-1100; 2 µg ml^−1^ and Alexa Fluor 594, cat. no. A-11005; 2 µg ml^−1^), and anti-goat (Alexa Fluor 488, cat. no. A-11055; 2 µg ml^−1^) were purchased from Thermo Fisher Scientific (Waltham, MA, USA).IL-1β (cat. no. AF-401-NA; 0.25 µg ml^−1^) was from R&D Systems, Inc. (Minneapolis, MN, USA). Elastin (cat. no. sc-58756; 1:500), CD-40 (cat. no. sc-1731;1:500), CD-206 (cat. no. sc-34577; 1:500), glyceraldehyde 3-phosphate dehydrogenase (GAPDH) (cat. no. sc-365062; 1:1000), and all HRP-conjugated secondary antibodies, i.e., anti-mouse (cat. no. sc-516102; 1:1000), anti-rabbit (cat. no. sc-2357; 1:1000), and anti-goat (cat. no. sc-2354; 1:1000) were from Santa Cruz Biotechnology (Santa Cruz, CA, USA). PVDF (cat. no. 1620177) membrane was purchased from Bio-Rad (Hercules, CA, USA). O.C.T. compound (cat. no. 23-730-571) was from Fisher Healthcare, (Houston, TX, USA). Bovine Serum Albumin (BSA) (cat. no. A30075) and non-fat dry milk powder (cat. no. M17200) were purchased from Research Products International Corp. (Mt. Prospect, IL, USA). Tween 20 (cat. no. M147) was from VWR Chemicals, LLC (Solon, OH, USA). Agarose (cat. no. BP-160) and mounting medium (cat. no. SP15) were purchased from Fisher Scientific (Fair Lawn, NJ, USA). DAPI (cat. no. F6057) and Nimbidiol [cat. no. SMB00209; molecular formula: ‘C_17_H_22_O_3_’, molecular weight: 274.35; purity: ≥ 95% (LC/MS-ELSD); IUPAC name: (4aS)-6,7-dihydroxy-1,1,4a-trimethyl-3,4,10,10a-tetrahydro-2H-phenanthren-9-one] were from Sigma-Aldrich (St. Louis, MO, USA).

### Animals

C57BL/6 J wild-type (WT) (stock no. 000664) and C57BL6/‐*Ins2*^*Akita*^/J type-1 diabetic (Akita) (stock no. 003548) male mice aged 10–14 weeks were purchased from the Jackson Laboratory (Bar Harbor, ME, USA). We chose male Akita mice because male mice spontaneously develop diabetes at the early age (five weeks) with high blood glucose levels while diabetes is less severe and more variable in female mice^99,100^. The mice were fed standard chow and water ad libitum. All animal experiments were conducted according to the protocols (Approval No. 20683, dated December 2, 2020) approved by the institutional animal care and use committee of the University of Louisville School of Medicine and conformed to the *Guide for the Care and Use of Laboratory Animals* published by the National Institutes of Health (NIH Publication, 2011), U.S.A. Animal studies were performed in compliance with the ‘ARRIVE’ guidelines. The mice were randomly segregated into four groups, viz. WT treated with saline [WT + saline], WT treated with Nimbidiol [WT + Nimbidiol], Akita treated with saline [Akita + Saline] and Akita treated with Nimbidiol [Akita + Nimbidiol]. WT and Akita mice were treated either with saline or with Nimbidiol (0.40 mg kg^−1^ d^−1^) using micro-osmotic pump for eight weeks. At the end of the experiment (eight weeks), mice were euthanized by using 2X tribromoethanol (TBE), and blood and kidney were collected.

### Micro-osmotic pump insertion

Under isoflurane anaesthesia, an incision was made along dorsal midline of the mice to form a subcutaneous pocket in the right flank using sterile forceps and blunt-tipped scissors. Saline or Nimbidiol-laden ALZET micro-osmotic pumps (Model 1004, DURECT Corporation, CA, USA) were implanted in the subcutaneous pocket for the delivery of Nimbidiol at the dose of 0.40 mg kg^−1^ d^−1^ for eight weeks.

### Measurements of body weight, blood glucose and glycated haemoglobin (HbA1c) levels, and α-glucosidase activity

Body weights of the mice were measured by an electronic balance (Model: EJ-1500, A&D, CA, USA). Blood glucose levels were obtained using a blood glucose meter (Ascensia Diabetes Care, NJ, USA). Blood levels of HbA1c were measured by a ‘Mouse Hemoglobin A1c (HbA1c) Assay Kit’ (cat. no. 80310, Crystal Chem, Inc., IL, USA). Plasma α-glucosidase activity was measured by a commercially available kit (cat. no. DAGD-100, BioAssay Systems, CA, USA).

### Glomerular filtration rate (GFR) measurement

Transcutaneous GFR was measured following the method described elsewhere^101^ with minor modifications. In brief, mice were anesthetized by isoflurane inhalation and then hair on the left dorsolateral part was shaved using a trimmer followed by topical application of Nair (Ewing, NJ). The NIC-Kidney device (Mannheim Pharma and Diagnostics, GmbH, Amtsgericht Mannheim, Germany) was gently fastened on the shaved area by an adhesive patch. FITC-sinistrin (7 mg/100 g b.w.) was injected into the femoral vein by a 32 gauge needle (TSK Laboratory, Japan) and monitored for next 2 h. MPD software (Mannheim Pharma and Diagnostics, GmbH, Amtsgericht Mannheim, Germany) was used to calculate GFR following the formula: GFR[µL/min/100 g b.w.] = 14,616.8 [µL/100 g b.w.]/t_1/2_(FITC-sinistrin) [min] as described previously^101^.

### Renal ultrasound

Ultrasonography was performed to assess the renal cortical blood flow as described earlier^102^. In brief, the mice were subjected to isoflurane anesthesia and placed to a warm platform at 37.5 °C. Left dorsolateral part of the mouse was depilated and an acoustic gel (Other-Sonic; Pharmaceutial Innovations, Newark, NJ) was applied on the shaved skin. Vevo 2100 system (VisualSonics, Toronto, ON, Canada) was used to perform Ultrasonography. Renal cortical blood vessels of the left kidney were scanned by the transducer, MS550D (22–55 MHz). Peak systolic velocity (PSV) and end-diastolic velocity (EDV) (mm/sec) in the renal cortical blood vessels were recorded in the Pulsed-Wave Doppler mode. Resistive index (RI) of the renal cortical blood vessels was determined by analyzing the exported cine loops.

### Laser Doppler flowmetry

Blood flow in the renal cortical vessels was determined by the Speckle Contrast Imager (Moor FLPI, Wilmington, DE, USA) as described previously^39^. Through a dorsal incision, the camera was focused on the kidney, aorta, renal artery and vein. Cortical flux units (No. of RBCs × velocity) were recorded as line traces.

### Isolation of RNA and semi-quantitative RT-PCR

Total RNA was isolated from kidney using Trizol reagent (cat. no. 15596-026, Invitrogen, Carlsbad, CA, USA) and reverse-transcribed using EasyScript cDNA Synthesis kit (cat. no. G234, MidSci, St. Louis, MO, USA) as per manufacturer′s instructions. cDNA was amplified by reverse transcriptase PCR using the GoTaq Hot Start Green Master Mix (cat. no. M5122, Promega, Madison, WI, USA) following manufacturer′s protocol. The PCR product was run on 1.5% agarose gel and the bands were visualized under UV light using a ChemiDoc XRS system (Bo-Rad, Hercules, CA). The expression of the gene was normalized with GAPDH. The band intensity was quantified by densitometry using ‘ImageJ’ software (Supplementary Information 1). The primer sequences (Invitrogen, Carlsbad, CA, USA) are mentioned in Table 1.

**Table 1:** Primer sequences used for semi-quantitative RT-PCR analysis.

	Forward	Reverse
Col IV	5′GACCACTATGCTTGAAGTGA3′	5′ACAGAAGGCCTTAGTAGTCT3′
Fibronectin	5′TTGTTCGGTGGAGTAGACCC3′	5′TTCAGGGAGGTTGAGCTCTG3′
Elastin	5′TGACAGTATAGGGCTGAGCA3′	5′GAGTTGTTGTGGGTGAGACA3′
MMP-9	5′CACACGACATCTTCCAGTACCA3′	5′TCATTTTGGAAACTCACACGCC3′
MMP-13	5′CAGTTGACAGGCTCCGAGAA3′	5′TTCACCCACATCAGGCACTC3′
CD40	5′ACTGATACCGTCTGTCATCC3′	5′CTTATCCTCACAGCTTGTCC3′
CD206	5′TGTTGATTGTTGATTGCCAC3′	5′ACCAGTGTAGCAGTGTTAAG3′
α-SMA	5′CTATGTGTGAAGAGGAAGACA3′	5′CATTCCAACCATTACTCCCT3′
E-cadherin	5′GGATCAGGACCAGGACTACG3′	5′AGCTTCTTGAATCGGTTGCC3′
TNF-α	5′GATCGGTCCCCAAAGGGATG3′	5′GGCTACAGGCTTGTCACTCG3′
IL-1β	5′TCCCTTTTCGTGAATGAGCAGA3′	5′GGAGGAAAACACAGGCTCTCTT3′
MCP-1	5′ACCACCTCAAGCACTTCTGTAG3′	5′TTAAGGCATCACAGTCCGAGTC3′
GAPDH	5′GTCAAGGCCGAGAATGGGAA3′	5′GGCCTCACCCCATTTGATGT3′

### Immunoblotting

An equal amount of protein was electrophoresed by sodium dodecyl sulphate- polyacrylamide gel electrophoresis (SDS-PAGE) and subsequently transferred onto the polyvinylidine difluoride (PVDF) membrane. 5% non-fat dry milk or BSA (for the detection of phosphorylated proteins) in TBST was used to block the membrane for 1 h at room temperature and standard immunoblotting was performed as described earlier^1^. GAPDH was used as a reference to normalize the expression of the protein. Densitometric analysis was performed to quantify the band intensity using ′ImageJ′ software (Supplementary Information 1).

### Hematoxylin and Eosin (H&E) staining

Neutral buffered formaldehyde-fixed, paraffin-embedded kidneys were cut at 5 μm thickness. The kidney sections were stained with hematoxylin (cat. no. 95057-844, VWR International, PA, USA) and eosin (cat. no. 26396-07, Electron Microscopy Sciences, PA, USA), and the histopathological changes were observed under an EVOS FL Automated System (Life Technologies, Inc., Grand Island, NY, USA).

### Periodic Acid-Schiff (PAS) staining

For PAS staining, 5-μm-thcik kidney sections were used to stain with ‘Periodic Acid Schiff Stain Kit’ (ES3400-IFU, Azer Scientific, PA, USA) according to the manufacturer′s instructions. Briefly, the sections were deparaffinized and hydrated to distilled water and incubated in Periodic Acid Solution for 10 min at room temperature. After rinsing in distilled water, the sections were immersed in Schiff’s Solution for 15 min. Slides were then rinsed in hot running tap water and distilled water followed by counterstaining with Mayer′s hematoxylin for 2‒3 min. After rinsing in running tap water, the slides were subjected to Bluing Reagent for 30 s. The slides were then rinsed in distilled water, dehydrated through graded alcohols, and mounted. The histopathological changes were evaluated under the EVOS FL Automated System (Life Technologies, Inc., Grand Island, NY, USA).

### Collagen staining

Collagen deposition in the kidney was analyzed by using a ‘Masson trichrome stain kit’ (cat. no. 87019, Richard-Allan Scientific, Kalamazoo, MI, USA). The kidney sections of 5 μm thickness were stained according to the manufacturer′s instructions. An EVOS FL Automated System (Life Technologies, Inc., Grand Island, NY, USA) was used to capture the images, and analyzed by ‘ImageJ’ software.

### Elastin staining

The kidney sections of 5 μm thickness were stained with an ‘Elastic stain kit’ (cat. no. 87017, Richard-Allan Scientific, Kalamazoo, MI, USA) as per manufacturer’s instructions. In brief, sections were deparaffinized and hydrated to deionized water followed by stained with ‘working elastic stain solution’ for 15–20 min. Excess stain was rinsed off with running tap water and the sections were decolorized with ‘working differentiating solution’. Slides were rinsed in tap water and then placed in ‘Sodium thiosulfate solution’ for 1 min. The sections were rinsed in deionized water and stained in ‘Van Gieson stain solution’ for 1 min. The sections were dehydrated and slides were mounted. The images were acquired using an EVOS FL Automated System (Life Technologies, Inc., Grand Island, NY, USA), and analyzed by ‘ImageJ’ software.

### Immunohistochemistry

The kidney cryosections of 5 μm thickness were fixed with freshly prepared 4% paraformaldehyde for 20 min. The sections were blocked with 1% BSA in PBS-T for 1 h at room temperature, and incubated overnight at 4 °C with appropriate primary antibody. Tissue sections were further incubated with corresponding Alexa Fluor 488 and/or 594-conjugated secondary antibodies (Invitrogen, Carlsbad, CA, USA) for 90 min at room temperature. An Olympus FluoView1000 laser scanning confocal microscope (B&B Microscope, Pittsburgh, PA, USA) was used to capture the images, and ‘ImageJ’ software was used to quantify the fluorescence intensity.

### Statistical analysis

Data are presented as mean ± standard deviation (SD) from 6 mice per group. The significance of difference between the means of different groups was determined by ANOVA followed by Tukey’s post hoc test. *P* < 0.05 was considered as significant.

## Supplementary Information

Supplementary Information.

## Data Availability

The data presented in this study are available on request from the corresponding author.
