# Supplementary material for: Glucosidase inhibitor, Nimbidiol ameliorates renal fibrosis and dysfunction in type-1 diabetes
Source: Sci Rep. 2022 Dec 15;12:21707. doi: 10.1038/s41598-022-25848-1 (PMC9755213; doi:10.1038/s41598-022-25848-1)
Supplement: Supplementary file 1 — Supplementary Information. [file 41598_2022_25848_MOESM1_ESM.pdf]

**Glucosidase inhibitor, Nimbidiol ameliorates renal fibrosis and dysfunction in type-1 diabetes**

Subir Kumar Juin, Sathnur Pushpakumar, Suresh C. Tyagi and Utpal Sen*

Department of Physiology, University of Louisville School of Medicine, Louisville, KY-40202

*Correspondence:
Utpal Sen
Department of Physiology
500 South Preston Street. HSC-A, Room 1115
University of Louisville School of Medicine
Louisville, KY 40202, USA
Tel: +1 502-852-2030
Fax: +1 502-852-6239
Email: u0sen001@louisville.edu

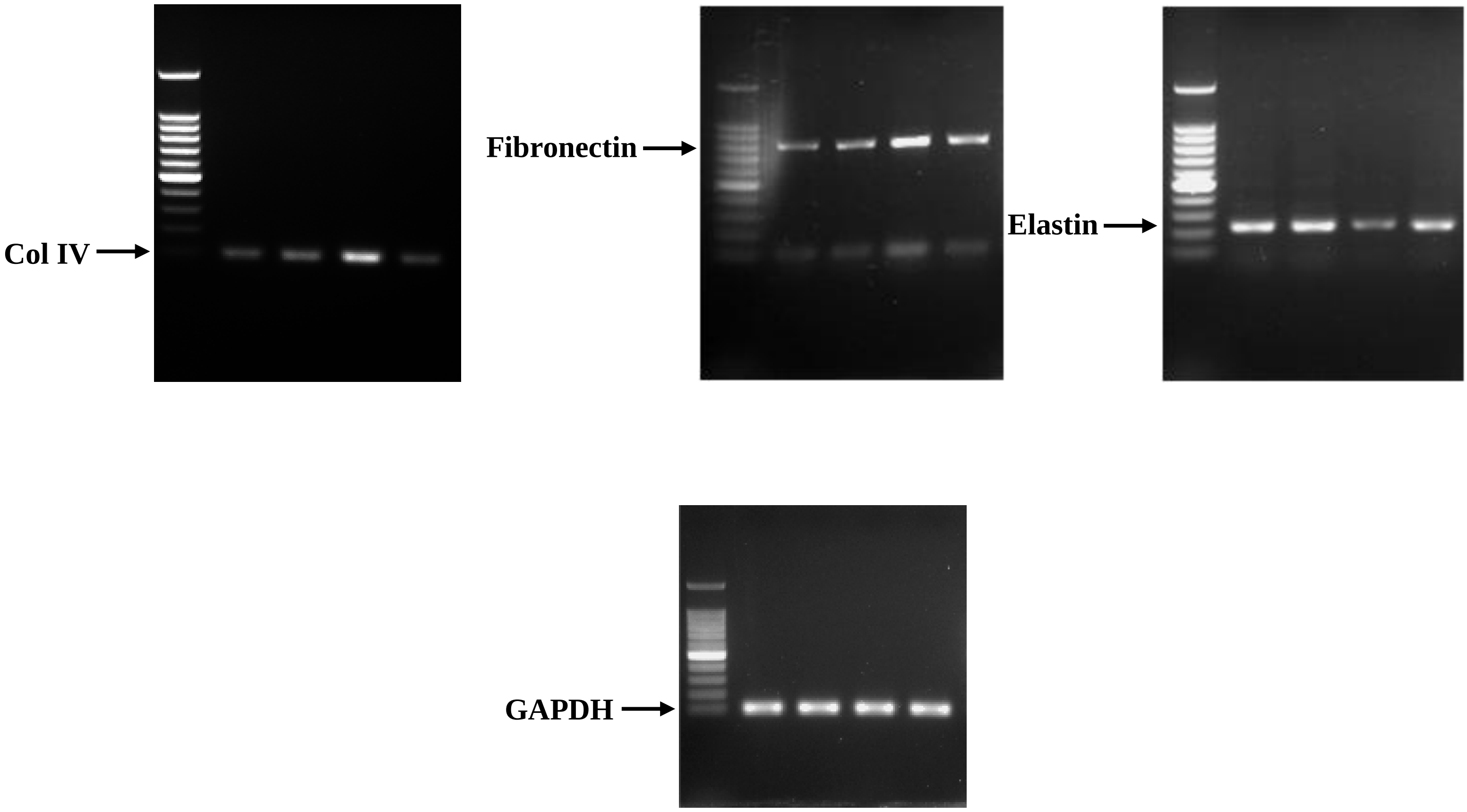


**Figure S1:** Uncropped images of Fig. 5A

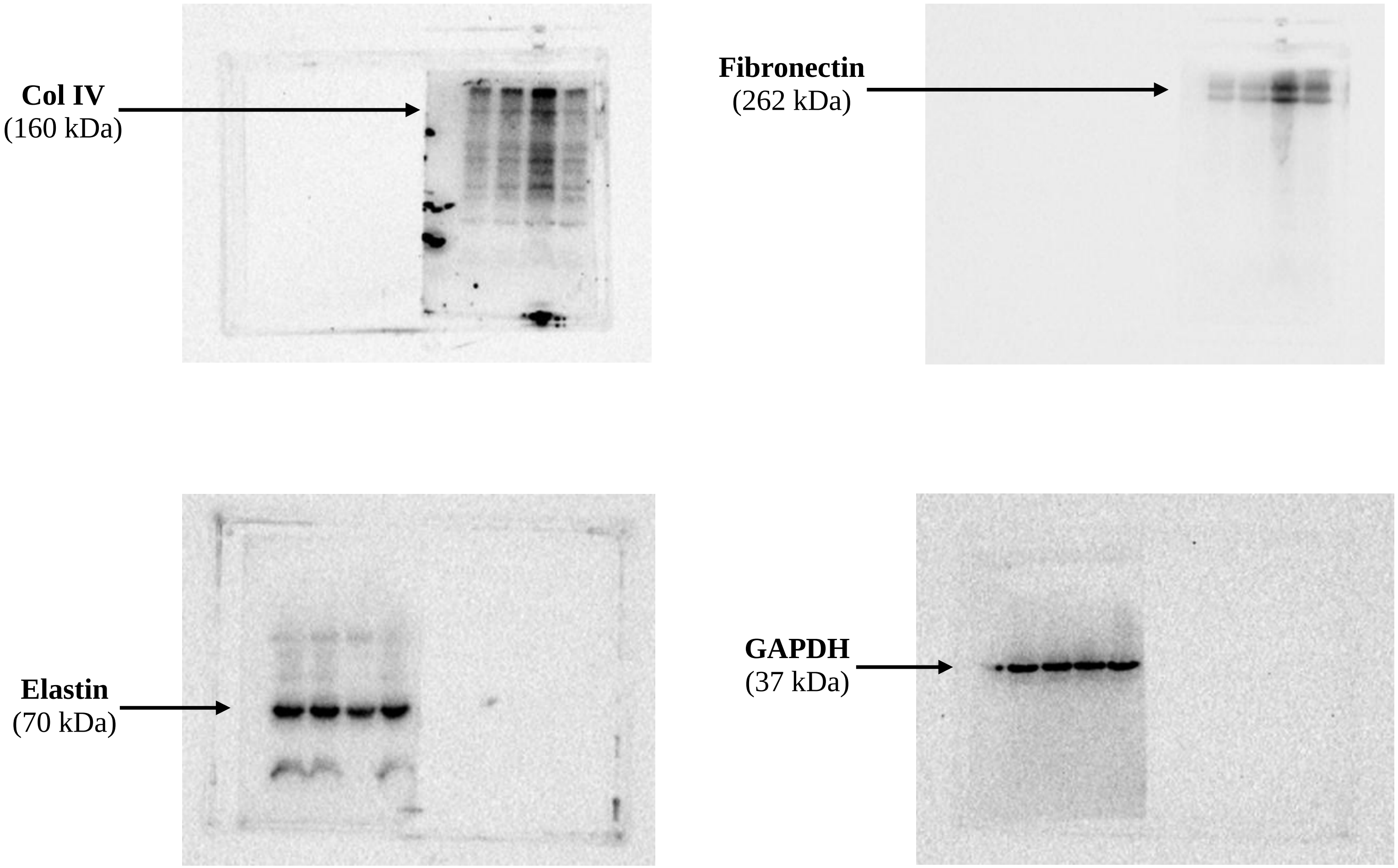


**Figure S2:** Uncropped images of Fig. 5B

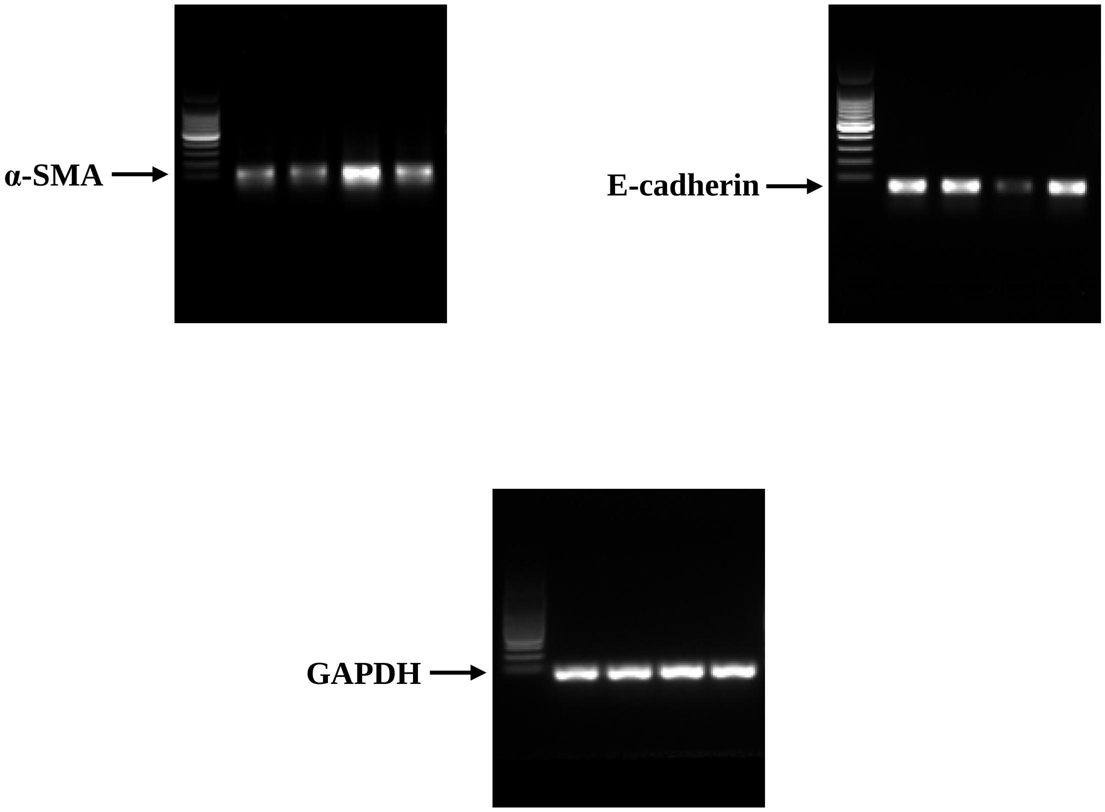


**Figure S3:** Uncropped images of Fig. 8A

**α-SMA**
(42 kDa)

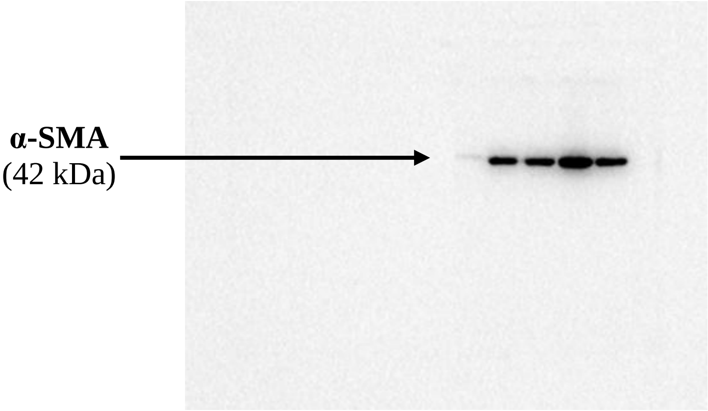

**E-cadherin**
(135 kDa)

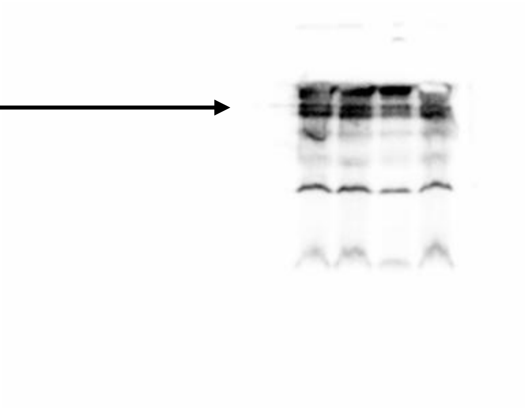

**GAPDH**
(37 kDa)

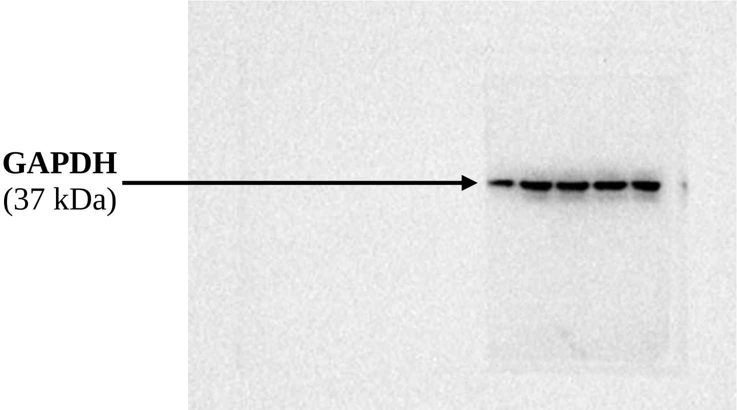

**Figure S4:** Uncropped images of Fig. 8B

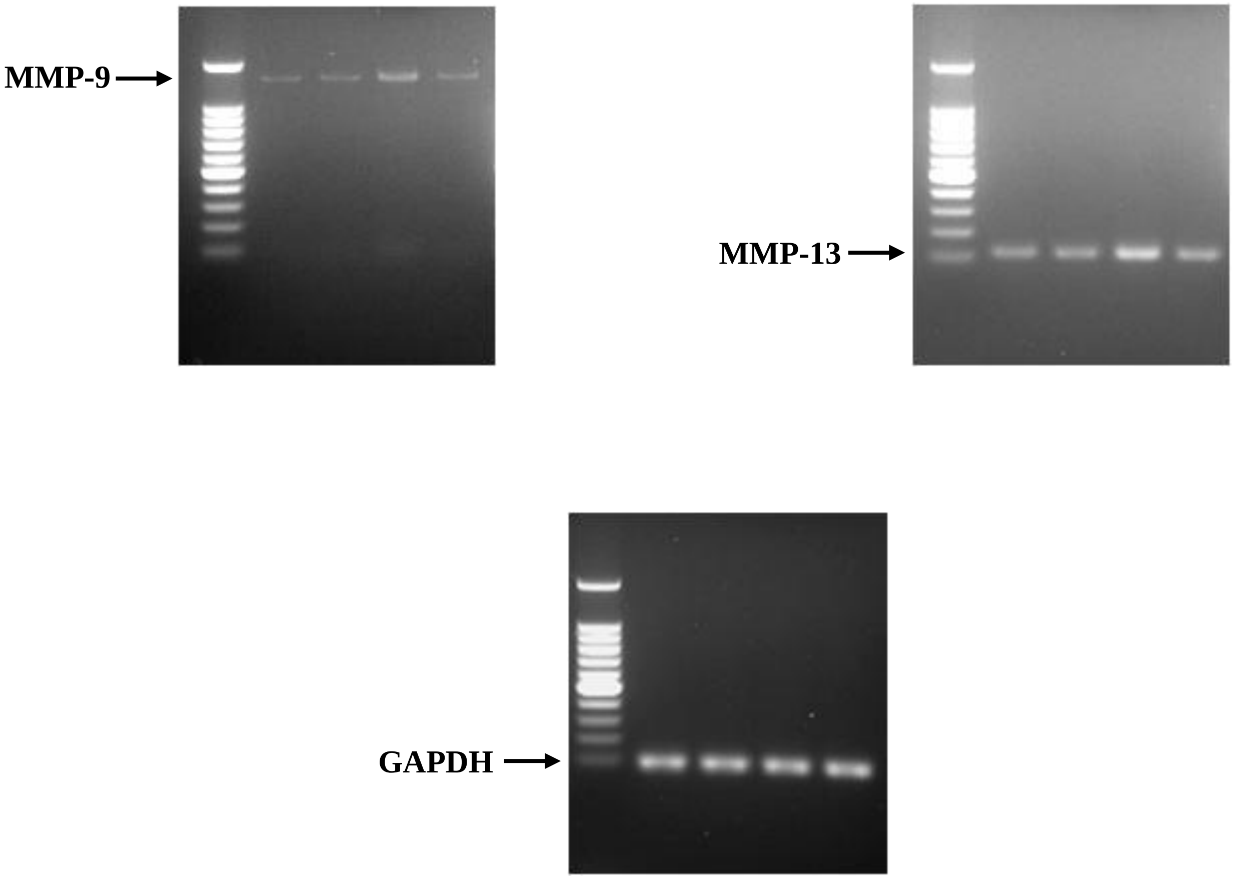


**Figure S5:** Uncropped images of Fig. 9A

**MMP-9**
(82 kDa)

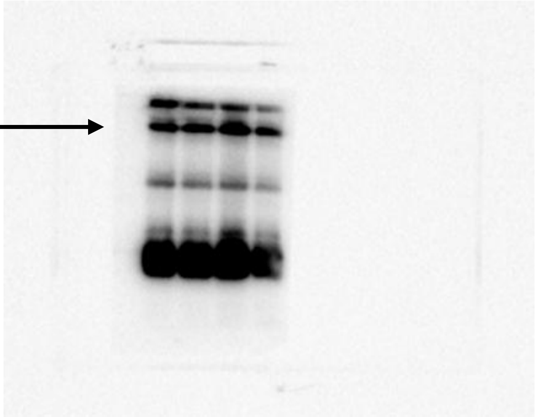

**MMP-13**
(50 kDa)

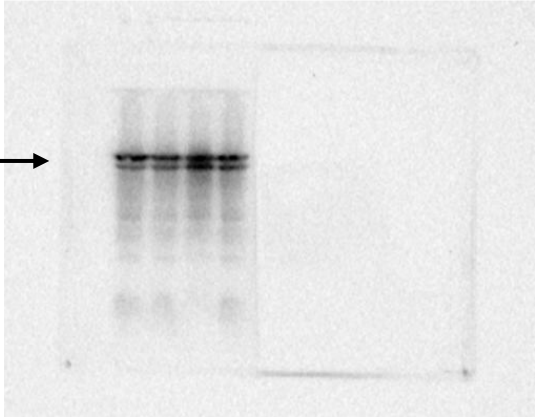

**GAPDH**
(37 kDa)

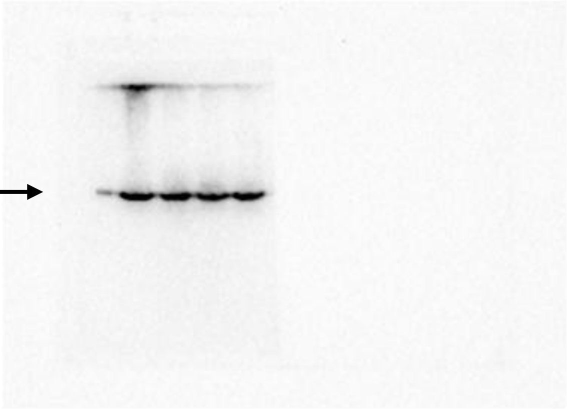

**Figure S6:** Uncropped images of Fig. 9B

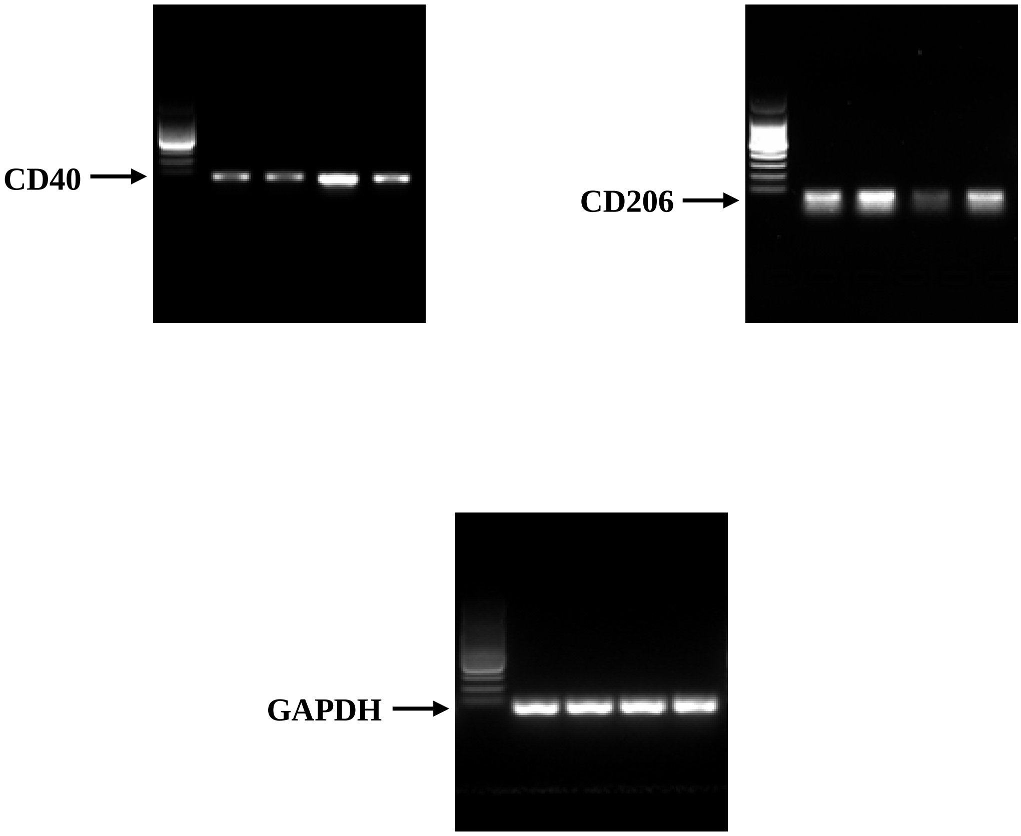


**Figure S7:** Uncropped images of Fig. 10A

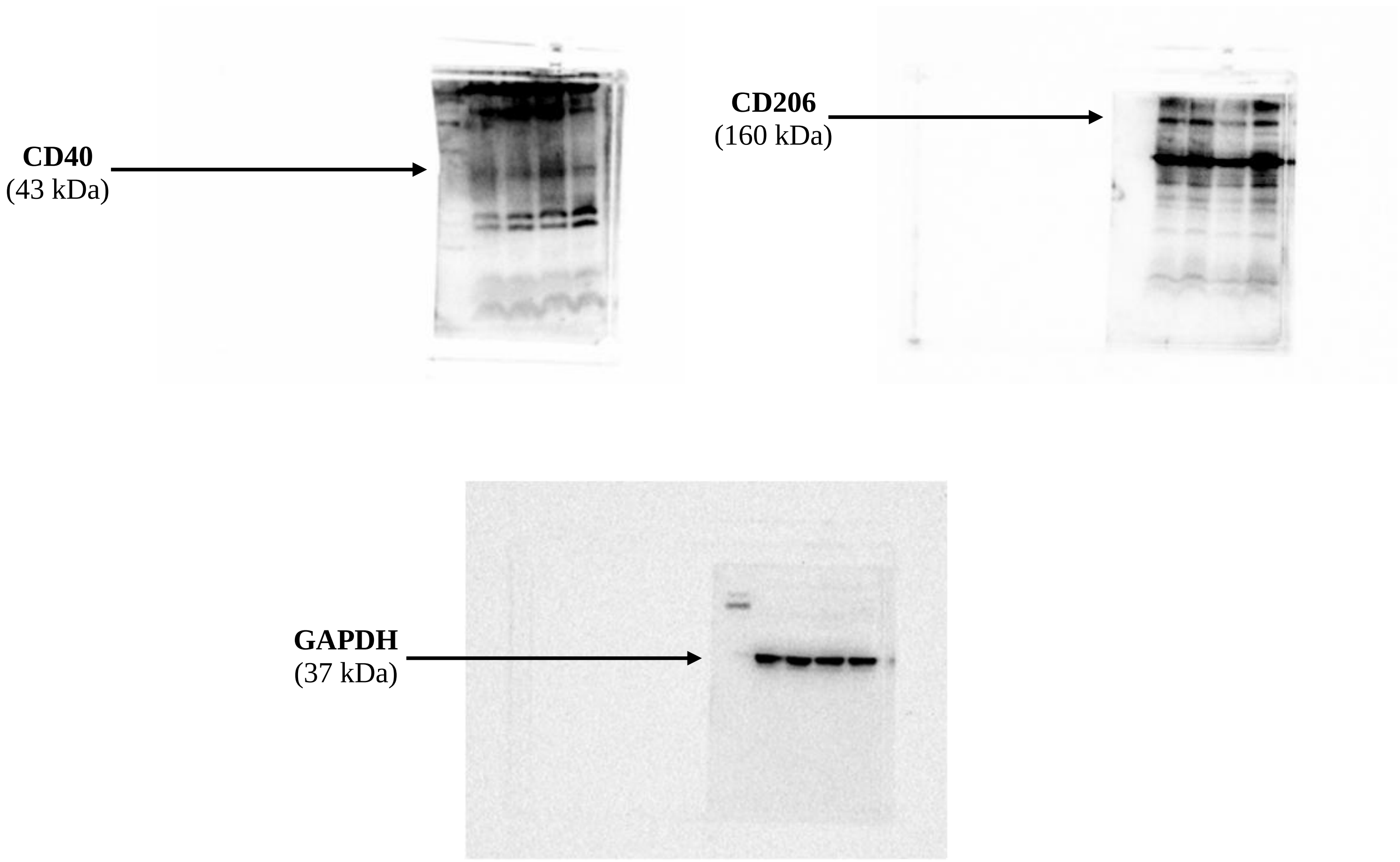


**Figure S8:** Uncropped images of Fig. 10B

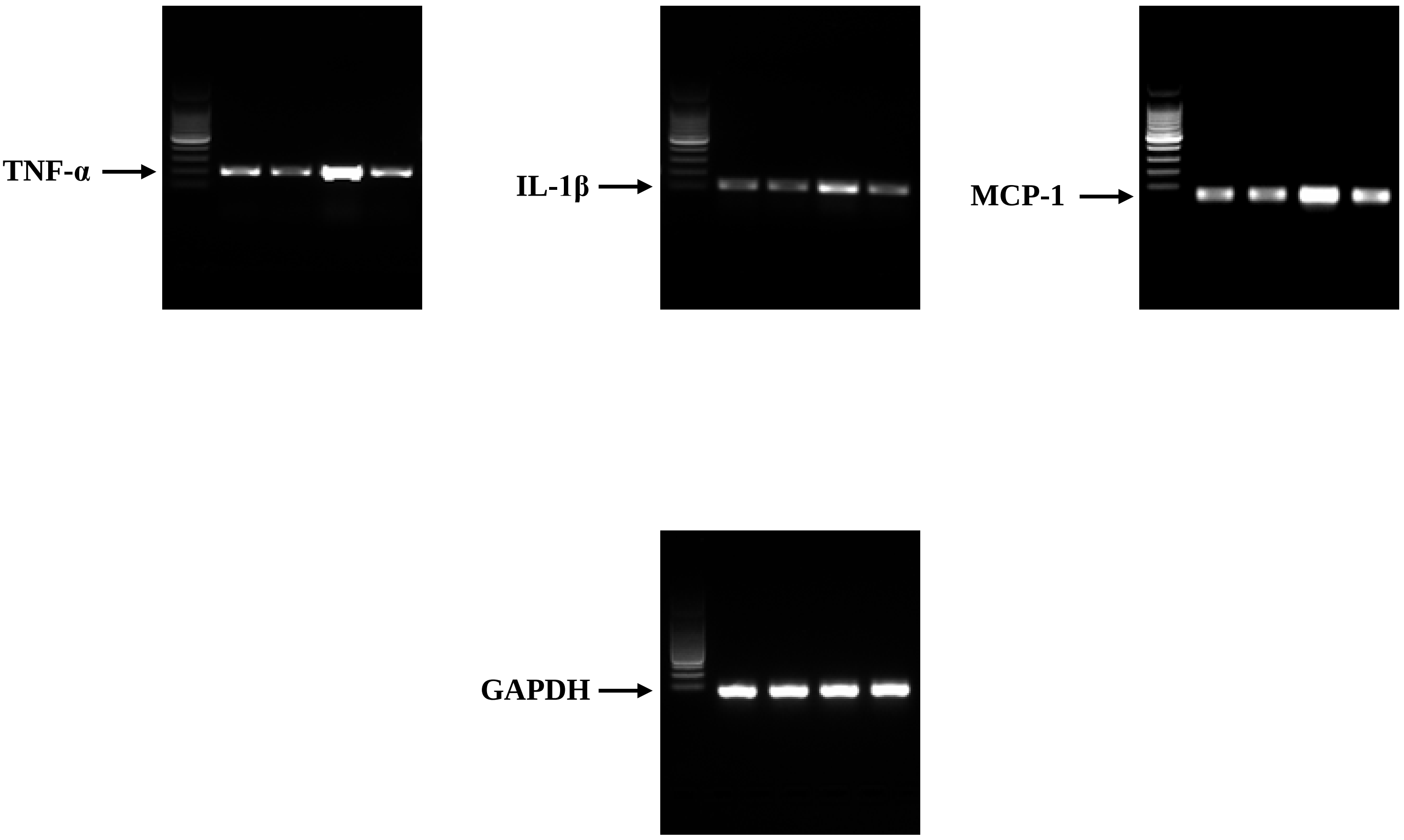


**Figure S9:** Uncropped images of Fig. 11A

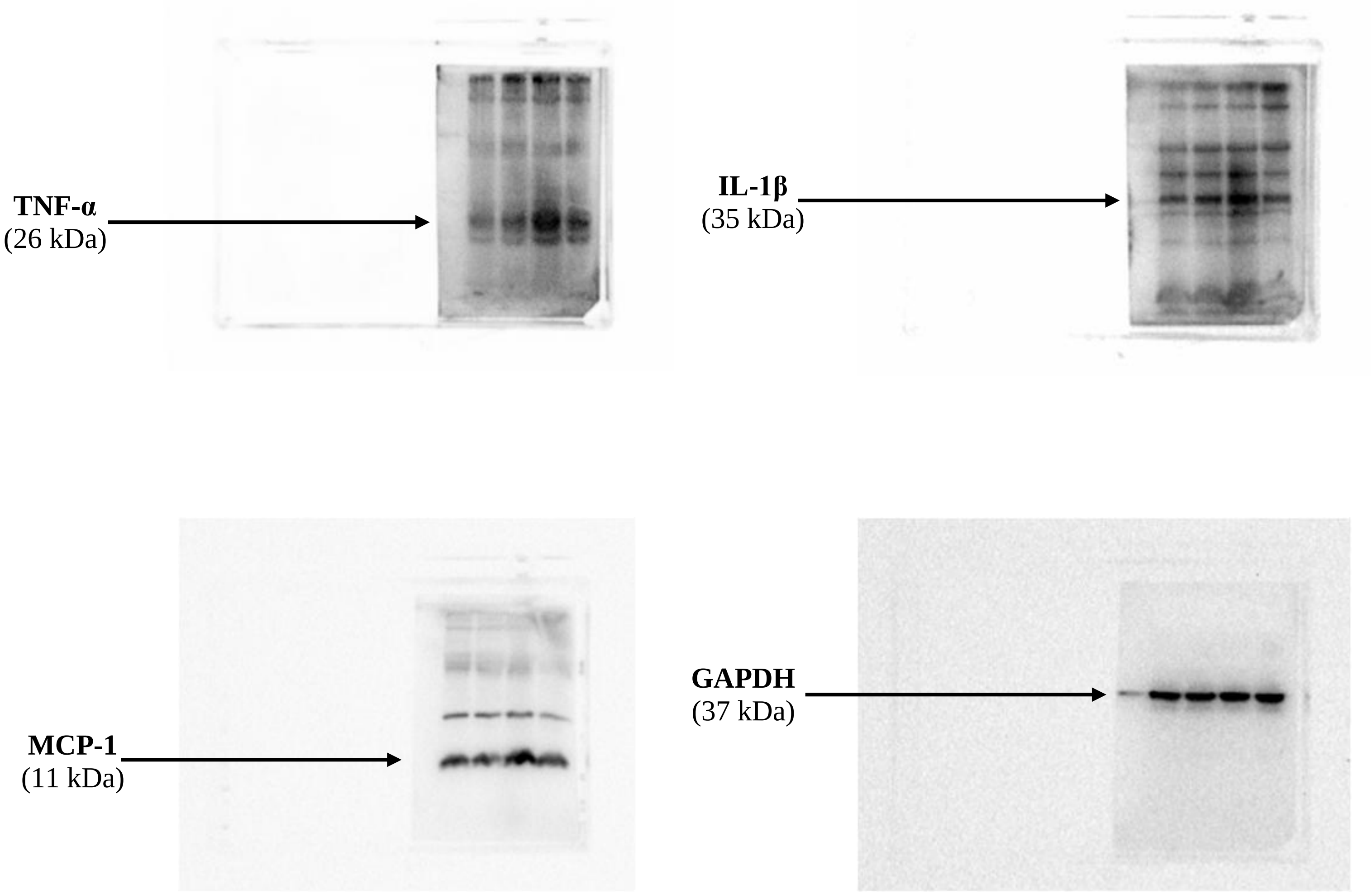


**Figure S10:** Uncropped images of Fig. 11B

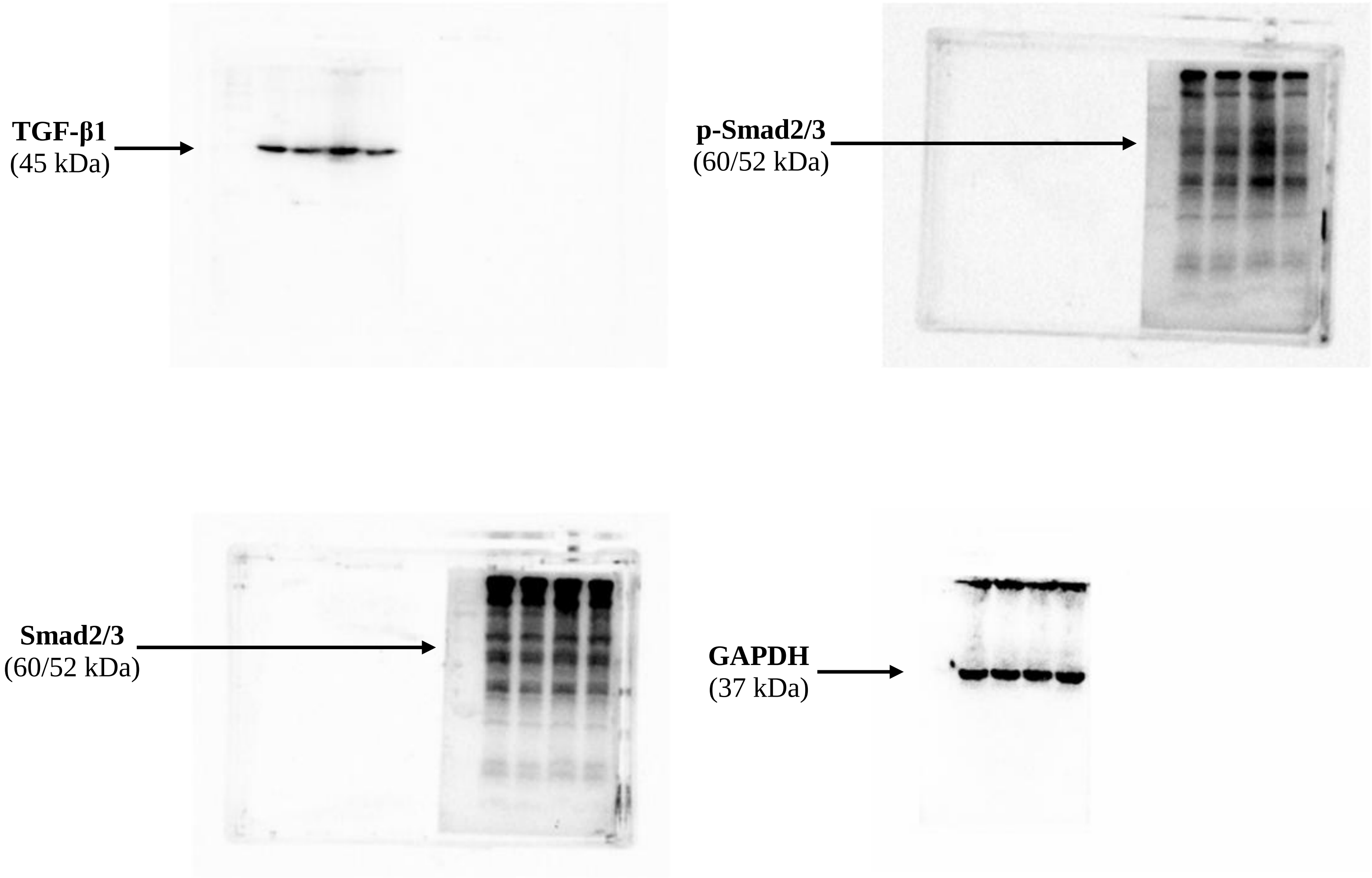


**Figure S11:** Uncropped images of Fig. 12A

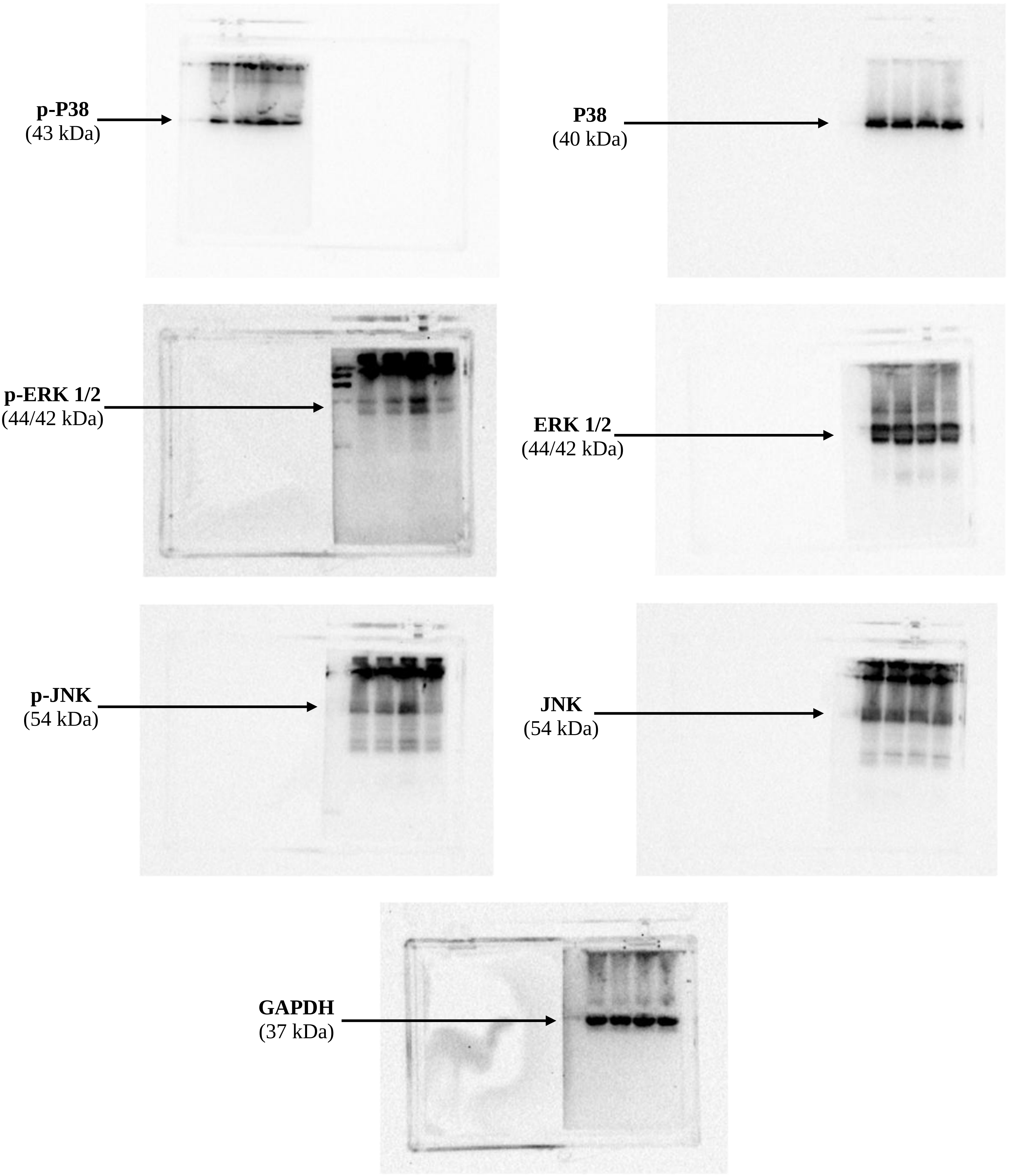


**Figure S12:** Uncropped images of Fig. 12B
